# The order Zoantharia Rafinesque, 1815 (Cnidaria, Anthozoa: Hexacorallia): supraspecific classification and nomenclature

**DOI:** 10.3897/zookeys.641.10346

**Published:** 2016-12-14

**Authors:** Martyn E. Y. Low, Frederic Sinniger, James Davis Reimer

**Affiliations:** 1 Lee Kong Chian Museum of Natural History, National University of Singapore, 2 Conservatory Drive, Singapore 117377, Republic of Singapore; 2 Tropical Biosphere Research Center, University of the Ryukyus, 3422 Sesoko, Motobu, Okinawa 905-0227, Japan; 3 Molecular Invertebrate Systematics and Ecology (MISE) Laboratory, Department of Marine and Environmental Sciences, Graduate School of Engineering and Science, University of the Ryukyus, 1 Senbaru, Nishihara, Okinawa 903‐0213, Japan; and Tropical Biosphere Research Center, University of the Ryukyus, 1 Senbaru, Nishihara, Okinawa 903-0213, Japan; 4 former address: Department of Marine and Environmental Sciences, Graduate School of Engineering and Science, University of the Ryukyus, 1 Senbaru, Nishihara, Okinawa 903-0213, Japan

**Keywords:** zoantharians, family-group, genus-group, taxonomy, historical literature

## Abstract

Many supraspecific zoantharian names have long and complicated histories. The present list is provided to advise researchers on the current state of supraspecific nomenclature of the zoantharians, particularly given the recent attention paid to the taxonomy, phylogeny, and biodiversity of this order. At the same time, several taxonomic issues brought to light by recent research are resolved. Details on the taxonomic and nomenclatural history of most groups are provided, along with appendices of invalid supraspecific names.

## Introduction

The Zoantharia are an order of Hexacorallia most closely related to the sea anemones (Actiniaria) (Kayal et al. 2013). They are found in most marine ecosystems. Despite their cosmopolitan distribution and high frequency in many ecosystems, compared to both Actiniaria and Scleractinia, research on this order has been relatively scant. This is due to both their high levels of intraspecific morphological variation (e.g. Burnett et al. 1997, Reimer et al. 2004, Ong et al. 2013) and the presence of incrustations in their body walls (for all zoantharian families except the Zoanthidae), which makes internal histological examination problematic (Reimer et al. 2010c). Taxonomic identification problems have been compounded by a general lack of utility in some traditionally used diagnostic characters (Sinniger et al. 2010a, Swain 2010). Thus, despite being common in many ecosystems (Karlson 1980), until very recently zoantharian taxonomy has been confused (Burnett et al. 1997).

However, recent molecular phylogenetic examination of zoantharians, combined with a reassessment of their taxonomy and diagnostic characters, has led to the group becoming somewhat ordered (Reimer et al. 2007a, 2008a, Reimer and Fujii 2010, Sinniger et al. 2010a, 2013, Fujii and Reimer 2011, 2013, Swain and Swain 2014; Swain et al. 2015), setting an example for the reorganization of other anthozoan groups. This follows in the footsteps of the database Biogeoinformatics of Hexacorallia (Fautin and Buddemeier 2008), which was established as a repository for taxonomic and nomenclatural information on the Hexacorallia (see also Fautin 2016).

The order Zoantharia (= Zoanthidea or Zoanthiniaria) is currently thought to be comprised of nine families—Abyssoanthidae Reimer & Fujiwara, in Reimer, Sinniger, Fujiwara, Hirano & Maruyama, 2007, Epizoanthidae Delage and Hérouard, 1910, Hydrozoanthidae Sinniger, Reimer & Pawlowski, 2010, Microzoanthidae Fujii & Reimer, 2011, Nanozoanthidae Fujii & Reimer, 2013, Neozoanthidae Herberts, 1972, Parazoanthidae Delage & Hérouard, 1901, Sphenopidae Hertwig, 1882, and Zoanthidae Rafinesque, 1815 (Daly et al. 2007: 143, 144; Hoeksema and Reimer 2013).

There is also a bioinformatics rationale for the compilation of such a taxonomic listing. As electronic name-lists of available and valid names become increasingly accessible, the need to link such lists with nomenclators of names will become an imperative. To this end, listings such as the present one can provide clear linkages between valid names and the huge mass of historical names (most of which are synonyms and some being potentially valid), which will effectively reduce the historical ‘synonymy load’ (Low and Reimer 2012a: 83) and provide a clear list of available supraspecific names of Zoantharia for future researchers.

### Additional notes for introductory text

All valid taxa are treated in the systematics section, with the taxon (genus and species) listed. Synonyms are also listed. Justification for these synonymies is given in the sections that follow (Appendices 1–3). We use the general term “zoantharian” to refer to species within this order, unless when quoting original text in which another term (usually “zoanthid”) was used.

### Statistics

A total of 16 family-group names are treated. Nine are considered to be valid, six are synonyms, and one is not referable to the order Zoantharia. A total of 102 names at the genus rank are treated. Of these, 28 names are considered to be valid, 38 to be synonyms, 19 are incorrect spellings, 18 are not referable to the order Zoantharia, and two—*Stephanidium* Hertwig, 1888, and *Triga* Gray, 1867—are of uncertain placement and validity (see Table 1).

## Systematics

### Phylum Cnidaria

Taxon classificationAnimalia

Hatschek, 1888

Cnidaria Hatschek, 1888 (in 1888–1891): 40.

#### Remarks.

See discussion on the nomenclature of the phylum in Fautin and Daly (2009: 315).

### Class Anthozoa

Taxon classificationAnimalia

Ehrenberg, 1831

Anthozoa Ehrenberg, 1831: 44.

### Subclass Hexacorallia

Taxon classificationAnimalia

Haeckel, 1896

Zoantharia de Blainville, 1830: 274.
Hexacorallia Haeckel, 1896: 217.

### Order Zoantharia

Taxon classificationAnimaliaZoantharia

Rafinesque, 1815

Zoanthia Rafinesque, 1815: 155.
Zoantharia Gray, 1832: 94.
Zoanthiniaria van Beneden, 1897: 150.
Zoanthidea Bourne, 1900: 58.

#### Diagnosis.

Anthozoans with body walls usually incrusted with sand and/or other detritus (except for the family Zoanthidae), tentacles always arranged in two rows or cycles. Majority of species are colonial.

#### Remarks.

Herein, we choose to use the name Zoantharia Rafinesque, 1815. Although Zoantharia Rafinesque, 1815, has identical spelling with the supraordinal name Zoantharia de Blainville, 1830, the latter name has fallen from common use—Hexacorallia Haeckel, 1896, being favoured. Furthermore, using the name Zoanthidea Bourne, 1900, can potentially cause confusion when used in the non-technical form “zoanthids” to denote members of the order, as it would be identical with the term “zoanthids” to denote members of the family Zoanthidae Rafinesque, 1815.

### Suborder Brachycnemina

Taxon classificationAnimaliaZoantharia

Haddon & Shackleton, 1891

Brachycneminae Haddon & Shackleton, 1891a: 626.

#### Diagnosis.

Zoantharians with the fifth mesenteries from the dorsal directive being incomplete.

#### Remarks.

—Recent molecular phylogenetic research indicates the two suborders of Zoantharia may not be monophyletics (e.g. Fujii and Reimer 2013). Three families are currently assigned to Brachycnemina Haddon & Shackleton, 1891.

### Neozoanthidae

Taxon classificationAnimaliaZoanthariaNeozoanthidae

Herberts, 1972

Neozoanthidae Herberts, 1972: 137.

#### Type genus.

*Neozoanthus* Herberts, 1972.

#### Gender.

Masculine.

#### Diagnosis.

Zooxanthellate, brachycnemic zoantharians with only partial incrustation in ectoderm, rarely extending to mesoglea, no incrustation around the top, oral ends of polyps (Herberts 1972).

#### Remarks.

A monogeneric and monospecific family-group. This taxon was originally defined as “Zoanthaires à arrangement mésentérique de type brachycnémique et à sphincter endodermique” (Herberts 1972: 137). However, recent work has further divided zoantharian sphincter muscles into ten different basic types; that of Neozoanthidae is discontiguous endodermal (Swain et al. 2015). Thus, the above diagnosis has been slightly modified from that of Herberts’ (1972).

Recent molecular phylogenetic work calls into the question the validity of this family, as molecular data derived from *Neozoanthus* specimens cluster within the Zoanthidae radiation (Reimer et al. 2011a). Incrustation does not extend to mesoglea and is only found in the ectoderm. The families Neozoanthidae and Zoanthidae are not synonymised herein, as the Neozoanthidae also has some phylogenetic relation with the Hydrozoanthidae and the relationships between these families will require additional research (Reimer et al. 2011a).

### Neozoanthus

Taxon classificationAnimaliaZoanthariaNeozoanthidae

Herberts, 1972

Neozoanthus Herberts, 1972: 137.

#### Type species.

*Neozoanthus tulearensis* Herberts, 1972, by monotypy.

#### Gender.

Masculine.

#### Diagnosis.

As for family above.

#### Remarks.

Molecular phylogenetic results (Reimer et al. 2011a) indicate that this genus appears to be very closely related to the genus *Isaurus* Gray, 1828 (a genus assigned to the family Zoanthidae). Three species included in this group are from the Indo-Pacific.

### Sphenopidae

Taxon classificationAnimaliaZoanthariaSphenopidae

Hertwig, 1882

Palythoidae (as “Palythoae”) Duchassaing de Fonbressin and Michelotti, 1860: 37.
Sphenopidae Hertwig, 1882: 120.

#### Type genus.

*Sphenopus* Steenstrup, 1856.

#### Gender.

Masculine.

#### Diagnosis.

Brachycnemic zoantharians with sand/detritus incrustation in the ectoderm and mesoglea.

#### Remarks.

The family-group name Sphenopidae Hertwig, 1882, is currently threatened by the senior subjective synonym Palythoidae Duchassaing de Fonbressin and Michelotti, 1860. To maintain widespread and current usage of the former name, and in accordance with Article 23.9.2 (ICZN 1999: 28, 29), an application is being prepared to request the International Commission on Zoological Nomenclature to suppress the senior subjective synonym Palythoidae Duchassaing de Fonbressin & Michelotti, 1860, in favour of Sphenopidae Hertwig, 1882. See additional discussion in Appendix 3.

The family-group Sphenopidae was established by Hertwig (1882), with the inclusion of only the type genus, *Sphenopus* Steenstrup, 1856. Two genera are currently assigned to Sphenopidae Hertwig, 1882.

### Sphenopus

Taxon classificationAnimaliaZoanthariaSphenopidae

Steenstrup, 1856

Sphenopus Steenstrup, 1856: 37.

#### Type species.

*Sabella marsupialis* Gmelin, 1791, by monotypy.

#### Gender.

Masculine.

#### Diagnosis.

Unitary (=solitary, non-colonial) brachycnemic zoantharians with sand/detritus incrustation in the ectoderm and mesoglea.

#### Remarks.

Distinct, large unitary polyps found embedded in sandy habitats with the oral disc clear of, and not attached to substrate (Soong et al. 1999, Reimer et al. 2012b, 2015b). Recent molecular work has also indicated that this this genus-group may form a clade with some *Palythoa* spp. (Reimer et al. 2012b, Fujii and Reimer 2016).

### Palythoa

Taxon classificationAnimaliaZoanthariaSphenopidae

Lamouroux, 1816

Palythoa Lamouroux, 1816: 359.
Corticifera Le Sueur, 1817: 178.
Polythoa Schweigger, 1819: 100 [incorrect spelling].
Polythea Gistel, 1848: 181 [incorrect spelling].
Gemmaria Duchassaing de Fonbressin and Michelotti, 1860: 55.
Polythoa (Corticithoa) Andres, 1883a: 521, 535–538.
Polythoa (Gemmithoa) Andres, 1883a: 521, 532, 533.
Parapalythoa Verrill, 1900: 560.
Protopalythoa Verrill, 1900: 562.
Haplotella Stechow, 1919: 853.

#### Type species.

*Palythoa* [*sic*] *stellata* Lamouroux, 1816 [= *Alcyonium mammillosum* Ellis, in Ellis & Solander, 1786], by subsequent designation by Haddon and Shackleton (1891b: 691) (see also discussion in Appendix 3).

#### Gender.

Feminine.

#### Diagnosis.

Colonial brachycnemic zoantharians with sand/detritus incrustation in the ectoderm and mesoglea. Currently, all species except for two described are zooxanthellate (Irei et al. 2015).

#### Remarks.

Specimens examined with a sphincter muscle of linear mesogleal type (Swain et al. 2015).

*Protopalythoa* was originally separated from *Palythoa* based on primarily polyp shape. Pax (1910) recommended merging *Palythoa* and *Protopalythoa*, and he defined three groups; “immersae”, “intermediae” and “liberae”, which have no taxonomic rank (see Ryland and Lancaster 2003). In 1923, Carlgren adopted this inclusive nomenclature. Similarly, Burnett et al. (1997) discussed the need to merge these genera but retained *Protopalythoa*. Ryland and Lancaster (2003) also discussed this issue and argued to keep *Protopalythoa* citing additional characters (zooxanthellate eggs in *Protopalythoa*; afterwards potentially observed in *Palythoa* spp. (Shiroma et al. 2010)). Herein, based on the several recent molecular studies (Sinniger et al. 2005, Reimer et al. 2006, 2007b, 2012a), we formally synonymise *Palythoa* and *Protopalythoa*, with *Palythoa* having priority.

Although at least 272 species-group names have been established in the genera *Palythoa* and *Protopalythoa* in the literature (see Fautin and Buddemeier 2008), with more research, these species-group names will likely be found to be synonyms—the result of high level of intraspecific morphological variation (see Burnett et al. 1994, Reimer et al. 2006, Hibino et al. 2014).

### Zoanthidae

Taxon classificationAnimaliaZoanthariaZoanthidae

Rafinesque, 1815

Zoanthidae (as “Zoanthia”) Rafinesque, 1815: 155.
Zoanthidae Gray, 1832: 95.

#### Type genus.

*Zoanthus* Lamarck, 1801.

#### Diagnosis.

Brachycnemic zoantharians with no or little sand/detritus encrustation. Continuous or divided marginal muscle, and zoanthinae larvae (Ryland and Lancaster 2003).

#### Remarks.

All members of this family are zooxanthellate (with endosymbiotic *Symbiodinium* spp.), and are found in sub-tropical and tropical waters. Authorship of the family-group name Zoanthidae should be attributed to Rafinesque (1815), and not Gray (1832, 1840) (see Low and Reimer 2012a). Three genera are currently assigned to Zoanthidae Rafinesque, 1815.

### Zoanthus

Taxon classificationAnimaliaZoanthariaZoanthidae

Lamarck, 1801

Zoanthus Cuvier, 1800: tables 9–10 [*nomen nudum*].
Zoanthus (as “Zoantha”) Lamarck, 1801: 363.
Mammillifera Le Sueuer, 1817: 177.
Actimastus Rafinesque, 1818: 271 [unnecessary replacement name]
Mamillifera Quoy & Gaimard, 1834: 169 [incorrect spelling]
Anthozoon Gistel, 1848: 181 [unnecessary replacement name]
Mammilifera Gistel, 1848: 181[incorrect spelling]
Polythoa (Mammithoa) Andres, 1883a: 521.
Polythoa (Mammothoa) Andres, 1883a: 533 [incorrect spelling].
Zoanthus (Rhyzanthus) Andres, 1883a: 538.

#### Type species.

*Actinia sociata* Ellis, 1768, by monotypy.

#### Gender.

Masculine.

#### Diagnosis.

Zooxanthellate, absence of mineral incrustations in the column/coenenchyme (excluding superficial surface attachments) brachycnemic zoantharians with smooth, usually erect polyps—except in *Zoanthus praelongus* Carlgren, 1954 (see discussion below)—with no endodermal invaginations. Mesogleal sphincter muscle with clear distal and proximal sections, mesogleal canal system but no encircling sinus (Duerden 1898, Swain and Swain 2014).

#### Remarks.

Species of the genus *Zoanthus* and *Acrozoanthus* have a double sphincter muscle, which is unique among zoantharians. Referred to in Swain et al. (2015) as discontiguous mesogleal type.

Despite over 150 species having been described in or assigned to this genus (see Fautin and Buddemeier 2008), many of the species-group names are likely to be synonyms—due to the high level of intraspecific morphological variation (see Burnett et al. 1995, 1997, Reimer et al. 2004). One species, *Zoanthus praelongus* Carlgren, 1954, has recumbent (non-erect) polyps (as in the genus *Isaurus*) but is clearly referable to *Zoanthus* (see Carlgren 1954, Muirhead and Ryland 1985, Reimer et al. 2008b).

### Acrozoanthus

Taxon classificationAnimaliaZoanthariaZoanthidae

Saville-Kent, 1893

Acrozoanthus Saville-Kent, 1893: 153, 154.

#### Type species.

*Acrozoanthus australiae* Saville-Kent, 1893, by monotypy.

#### Gender.

Masculine.

#### Diagnosis.

Zooxanthellate, absence of mineral incrustations in the column/coenenchyme (excluding superficial surface attachments) brachycnemic zoantharians inhabiting the outside of eunicid worms, with a ‘budding’ method of asexual reproduction (Reimer et al. 2011b).

#### Remarks.

Specimens examined with a discontiguous mesogleal sphincter muscle (Swain et al. 2015).

The genus *Acrozoanthus* has a long and complicated taxonomic history. Reimer et al. (2011b) discussed that “despite its very similar appearance, *Acrozoanthus australiae* was placed into a genus separate from *Zoanthus* due to the presence of an axial skeleton (Saville-Kent 1893). Later it was shown that this skeleton was in fact a result of habitat preference as *Acrozoanthus* inhabits the outside of eunicid worm tubes, and the genus was subsequently merged back again into the genus *Zoanthus* (Haddon 1895). Subsequent to its original description, this species was not mentioned in literature again until its rediscovery by Ryland (1997), based on examination of a single specimen. Further work by Ryland and co-workers described the nematocysts of *Acrozoanthus australiae* (Ryland et al. 2004) and also an unusual ‘budding’ method of asexual reproduction (Ryland 1997), which was theorized to potentially confirm the placement of this species in its own genus”.

Molecular phylogenetic data indicate that *Acrozoanthus* is within the *Zoanthus* (see Reimer et al. 2011b) monophyly. However, due to a need for more detailed investigations, we refrain from synonymising *Acrozoanthus* with *Zoanthus*.

### Isaurus

Taxon classificationAnimaliaZoanthariaZoanthidae

Gray, 1828

Isaura Lamouroux, in Audouin, Bourdon, de Candolle, d’Aubebard de Férussac, Deshayes, Deslongchamps, É. Geoffroy Saint-Hilaire, I. Geoffroy Saint-Hilaire, Guérin, Guillemin, de Jussieu, Kunth, Delafosse, Lamouroux, Latreille, Prévost, Richard and Bory de Saint-Vincent, 1826: 23 [*nomen oblitum*].
Isaurus Gray, 1828: 8 [*nomen protectum*]
Isaura Agassiz, 1845: 14 [unjustified emendation].
Antinedia Duchassaing de Fonbressin & Michelotti, 1866: 136.
Pales Gray, 1867: 8.
Panceria Andres, 1877: 221–226.
Polythoa (Monothoa) Andres, 1883a: 521.
Zoanthus (Monanthus) Andres, 1883a: 538, 549, 541, 543.
Isaua Volpi & Benvenuti, 2003: 72 [incorrect spelling].

#### Type species.

*Isaurus tuberculatus* Gray, 1828, by monotypy.

#### Gender.

Masculine.

#### Diagnosis.

Zooxanthellate, absence of mineral incrustations in the column/coenenchyme (excluding superficial surface attachments) brachycnemic zoantharians with recumbent (non-erect) polyps. Often have tubercules (raised bumps = endodermal invagination) on the outer surface of polyps, except in *Isaurus maculatus* Muirhead & Ryland, 1985, which has a smooth polyp surface.

#### Remarks.

Specimens examined with an orthogonally-reticulate mesogleal sphincter muscle (Swain et al. 2015).

Ryland and Lancaster (2003) recognised only three valid species in this genus, although 22 species have been described in or assigned to this genus.

### Suborder Macrocnemina

Taxon classificationAnimaliaZoantharia

Haddon & Shackleton, 1891

Macrocneminae Haddon & Shackleton, 1891a: 626.

#### Diagnosis

Zoantharians with the fifth mesenteries from the dorsal directive being complete.

#### Remarks.

As stated above, the two suborders of Zoantharia appear not to be monophyletic (Sinniger et al. 2005). Three families are currently assigned to Macrocnemina Haddon & Shackleton, 1891.

### Epizoanthidae

Taxon classificationAnimaliaZoanthariaEpizoanthidae

Delage & Hérouard, 1901

Mardoellidae Danielssen, 1890: 116, 117 [*nomen oblitum*].
Epizoanthinae Delage & Hérouard, 1901: 664 [*nomen protectum*].

#### Type genus.

*Epizoanthus* Gray, 1867.

#### Diagnosis.

Macrocnemic zoantharians with a simple mesogleal muscle (Sinniger and Häussermann 2009: 26).

#### Remarks.

The family-group Epizoanthidae (as “Epizoanthinae”) was established by Delage and Hérouard (1901: 664, 665) as monotypic, with the inclusion of only the type genus. Three genera are currently assigned to Epizoanthidae.

Recent molecular and morphological studies (e.g. Swain et al. 2015) suggest that this diagnosis should be revised following a complete revision of the suborder. Currently, molecular signatures such as those suggested in Sinniger et al. (2008) and in Sinniger et al. (2013) appear efficient to distinguish macrocnemic genera.

### Epizoanthus

Taxon classificationAnimaliaZoanthariaEpizoanthidae

Gray, 1867

Sidisia Gray, 1858: 489 [suppressed in Opinion 1689, ICZN 1992].
Epizoanthus Gray, 1867: 237 [conserved in Opinion 1689, ICZN 1992].
Carolia Gray, 1867: 239 [invalid name, junior homonym].
Polythoa (Endeithoa) Andres, 1883a: 521, 531.
Verrillia Andres, 1883a: 520, 545.
Zoanthus (Corticanthus) Andres, 1883a: 538, 541.
Mardoell Danielssen, 1890: 117–126.
Marodellia Blanchard, 1893: 130 [unjustified emendation]
Mardoella Bell, 1906: 762 [incorrect spelling].
Lirrevia Delphy, 1939: 270.

#### Type species.

*Dysidea papillosa* Johnston, 1842, by monotypy (see also Opinion 1689, ICZN 1992).

#### Gender.

Masculine.

#### Diagnosis.

As for family but readily distinguishable from *Palaeozoanthus* by the presence of non-fertile micromesenteries (Sinniger and Häussermann 2009: 26).

#### Remarks.

Most species with reticulate mesogleal muscle, *Epizoanthus illoricatus* Tischbierek, 1930 with simplified mesogleal sphincter muscle (Swain et al. 2015).

As discussed in Sinniger and Häussermann (2009: 26), this genus is characterised by the “[p]olyps usually strongly encrusted with sand particles. Species found on rocky substrata or gastropod shells often inhabited by pagurids; some cases of free-living species reported (*Epizoanthus lindhali*, *Epizoanthus vagus*). In colonial species, polyps linked by stolons or, in pagurid-associated species, by a continuous coenenchyme. No symbioses with *Symbiodinium* zooxanthellae”.

### Palaeozoanthus

Taxon classificationAnimaliaZoanthariaEpizoanthidae

Carlgren, 1924

Palaeozoanthus Carlgren, 1924: 470–473.

#### Type species.

*Paleozoanthus reticulatus* Carlgren, 1924, by original description and monotypy.

#### Gender.

Masculine.

#### Diagnosis.

Macrocnemic zoantharians with a simple mesogleal muscle, and fertile micromesenteries.

#### Remarks.

This genus is monospecific, and is comprised of the type species *Paleozoanthus reticulatus*, which has not been encountered since it was first described. Due to similarities in sphincter muscles (Swain et al. 2015) further studies are needed to determine if this genus corresponds to *Terrazoanthus*.

### Thoracactis

Taxon classificationAnimaliaZoanthariaEpizoanthidae

Gravier, 1918

Thoracactis Gravier, 1918: 12.
Thoracactus Walsh, 1967: 49 [unjustified emendation and junior objective synonym].
Toracactis Herberts, 1972: 80 [incorrect spelling].

#### Type species.

*Thoracactis topsenti* Gravier, 1918, by monotypy.

#### Gender.

Masculine.

#### Diagnosis.

Rudimentary sphincter muscles, azooxanthellate, with no mesogleal channels or lacunae, found on hexactinellid sponges.

#### Remarks.

This is a monospecific genus comprised of only the type species *Thoracactis topsenti*. In describing *Thoracactis topsenti*, Gravier (1918) incorrectly identified it as an actinian (anemone) based on the lack of zooxanthellae, channels, gaps, or cell islets. Although currently placed in the family Epizoanthidae, based on the current understanding of the type species, this genus is referable to the family Parazoanthidae, although an examination of the type material will be necessary to confirm this (see Reimer et al. 2010a: 158).

### Hydrozoanthidae

Taxon classificationAnimaliaZoanthariaHydrozoanthidae

Sinniger, Reimer & Pawlowski, 2010

Hydrozoanthidae Sinniger, Reimer & Pawlowski, 2010: 60.

#### Type genus.

*Hydrozoanthus* Sinniger, Reimer & Pawlowski, 2010.

#### Diagnosis.

“This family is erected to group former Parazoanthidae species sharing specific insertions and deletions in mt-16S rDNA, especially in the V5 region (as defined in Sinniger et al. 2005) of this gene” and “Phylogenetically, species in this family are more closely related to brachycnemic zoanthids (especially from the genus *Palythoa*) than to other parazoanthids.” (Sinniger et al. 2010a: 61).

#### Remarks.

“This family groups several tropical and sub-tropical macrocnemic zoanthids; including species associated with hydrozoans and also several other non-hydrozoan associated species.” (Sinniger et al. 2010a: 61). This family is currently comprised of two genera— *Hydrozoanthus* Sinniger, Reimer & Pawlowski, 2010, and *Terrazoanthus* Reimer & Fujii, 2010.

### Hydrozoanthus

Taxon classificationAnimaliaZoanthariaHydrozoanthidae

Sinniger, Reimer & Pawlowski, 2010

Hydrozoanthus Sinniger, Reimer & Pawlowski, 2010: 60.

#### Type species.

*Parazoanthus tunicans* Duerden, 1900, by original designation.

#### Gender.

Masculine.

#### Diagnosis.

A hydrozoanthid associated with hydrozoans.

#### Remarks.

Examined species with branchiform endodermal sphincter muscle (Swain et al. 2015).

### Terrazoanthus

Taxon classificationAnimaliaZoanthariaHydrozoanthidae

Reimer & Fujii, 2010

Terrazoanthus Reimer & Fujii, 2010: 20.

#### Type species.

*Terrazoanthus onoi* Reimer & Fujii, 2010, by original designation.

#### Gender.

Masculine.

#### Diagnosis.

This genus is characterised by being a member of the Hydrozoanthidae that is found on rocky substrates (as opposed to being obligate symbionts with hydrozoans). Some species in this genus are also brightly coloured (see Reimer and Fujii 2010: 20).

#### Remarks.

Sphincter muscle transitional, with distal half mesogleal and proximal half endodermal, with encrustations to endodermal surface of mesoglea (Swain and Swain 2014) (=meso-endo transitional [Swain et al. 2015]), although *Terrazoanthus minutus* (Duerden, 1898) has a simplified mesogleal sphincter muscle (Swain et al. 2015).

Described species currently referable to the genus *Terrazoanthus* are mainly from the East Pacific, with *Terrazoanthus minutus* from the Caribbean, and it is likely that several more undescribed species exist in the Atlantic (see Reimer et al. 2010a, 2012a) and in the Central Indo-Pacific region (Reimer et al. 2014b).

The diagnosis of *Terrazoanthus* is in need of revision with the placement of *Terrazoanthus patagonichus* (Carlgren, 1898) into this genus by Swain et al. (2015) as this species is associated with hydroids (McMurrich 1904).

### Microzoanthidae

Taxon classificationAnimaliaZoanthariaMicrozoanthidae

Fujii & Reimer, 2011

Microzoanthidae Fujii & Reimer, 2011: 420, 421.

#### Type genus.

*Microzoanthus* Fujii and Reimer, 2011.

#### Diagnosis.

As discussed in Fujii and Reimer (2011: 421), this family is characterised by “[c]olonies attached to bottom side (downward facing side) of dead coral rubble, asperous stones, inside narrow cracks, or occasionally on dead coral rubble on muddy seafloor. Azooxanthellate, macrocnemic. Polyps connected by narrow stolon or solitary. Sand particles encrusted in column. Irregularly sized sand particles encrusted into ectoderm. Tentacles two to three times as long as expanded oral disc diameter. Edge of oral disc shaped in regular, repeating zig-zagged pattern”.

#### Remarks.

This is a monotypic family and comprises only the genus *Microzoanthus* Fujii & Reimer, 2011, with two species.

### Microzoanthus

Taxon classificationAnimaliaZoanthariaMicrozoanthidae

Fujii & Reimer, 2011

Microzoanthus Fujii & Reimer, 2011: 420, 421.

#### Type species.

*Microzoanthus occultus* Fujii & Reimer, 2011, by original designation.

#### Gender.

Masculine.

#### Diagnosis.

As for family (Fujii and Reimer 2011). Encircling sinus present just beneath ectodermal surface (Swain and Swain 2014).

#### Remarks.

Examined specimens with spindly-cteniform endodermal sphincter muscle (Swain et al. 2015).

Currently two species, reported only from the Pacific Ocean (Fujii and Reimer 2013) and the Red Sea (Reimer et al. 2014c).

### Nanozoanthidae

Taxon classificationAnimaliaZoanthariaNanozoanthidae

Fujii & Reimer, 2013

Nanozoanthidae Fujii & Reimer, 2013: 512.

#### Type genus.

*Nanozoanthus* Fujii & Reimer, 2013.

#### Diagnosis.

“Well developed polyps connected by narrow stolon. Mineral particles encrusted in column from aboral end to the edge of the oral disc. Irregularly sized sand particles encrusted into ectoderm and slightly into mesoglea. Zig-zagged, white-colored pattern following outside edge of oral disc. Macrocnemic mesenterial arrangement. Sphincter muscle mesogleal. No lacunae or ring sinus. Zooxanthellate. Mitochondrial cytochrome oxidase subunit I and 16S ribosomal DNA sequences significantly differ from all other known zoanthid genera (Fig. 1, 2).” (Fujii and Reimer 2013).

#### Remarks.

A monogeneric family. Molecular data position this family in an intermediate position between the Brachycnemina and Macrocnemina, although currently it is placed within Macrocnemina (Fujii and Reimer 2013).

### Nanozoanthus

Taxon classificationAnimaliaZoanthariaNanozoanthidae

Fujii & Reimer, 2013

Nanozoanthus Fujii & Reimer, 2013: 512–515.

#### Type species.

*Nanozoanthus harenaceus* Fujii & Reimer, 2013, by original designation.

#### Gender.

Masculine.

#### Diagnosis.

As for family above.

#### Remarks.

This is a monospecific genus currently, with specimens reported from southern Japan, Western Australia, and the Red Sea (Reimer et al. 2015a). However, molecular data indicate that the European species *Isozoanthus sulcatus* (Gosse, 1859) likely also belongs to this genus (Fujii and Reimer 2013).

### Parazoanthidae

Taxon classificationAnimaliaZoanthariaParazoanthidae

Delage & Hérouard, 1901

Savaliidae (as “Savalini”) Nardo, 1844: 433.
Bergiidae Verrill, 1865a: 147 [*nomen oblitum*].
Gerardiidae Verrill, 1865a: 148.
Savagliidae Brook, 1889: 51, 74, 79.
Parazoanthidae Delage & Hérouard, 1901: 665 [*nomen protectum*].
Savaliidae Poche, 1914: 104.
Heterozoanthidae Pax & Müller, 1956: 3.

#### Type genus.

*Parazoanthus* Haddon & Shackleton, 1891.

#### Diagnosis.

As discussed in Sinniger and Häussermann (2009: 28), this family “[…] traditionally groups macrocnemic zoanthids possessing an endodermal sphincter. Member species are frequently associated with other organisms, which are used as substrata”. Excludes species that form monophyly with Brachycnemina (Sinniger et al. 2010a).

#### Remarks.

Precedence of Bergiidae Verrill, 1865, and *Bergia* Duchassaing de Fonbressin & Michelotti, 1860, and respectively Parazoanthidae Delage & Hérouard, 1901, and *Parazoathus* Haddon & Shackleton, 1891a, was reversed in Low and Reimer (2011a). In accordance with Article 23.9.2 (ICZN 1999: 28, 29), an application is being prepared to request that the International Commission on Zoological Nomenclature suppress the senior subjective synonyms Savaliidae Nardo, 1844, Gerardiidae Verrill, 1865, and Savagliidae Brook, 1889, in favour of Parazoanthidae Delage & Hérouard, 1901, to maintain current and widespread usage. See additional discussion in Appendix 3.

In addition to the type genus, *Parazoanthus* Haddon & Shackelton, 1891, twelve other valid genera are currently assigned to the family Parazoanthidae: *Antipathozoanthus* Sinniger, Reimer & Pawlowski, 2010, *Bergia* Duchassaing de Fonbressin & Michelotti, 1860, *Bullagummizoanthus* Sinniger, Ocaña & Baco, 2013, *Corallizoanthus* Reimer, in Reimer, Nonaka, Sinniger & Iwase, 2008, *Hurlizoanthus* Sinniger, Ocaña & Baco, 2013, *Isozoanthus* Carlgren, in Chun, 1903, *Kauluzoanthus* Sinniger, Ocaña & Baco, 2013, *Kulamanamana* Sinniger, Ocaña & Baco, 2013, *Mesozoanthus* Sinniger & Häussermann, 2009, *Savalia* Nardo, 1844, *Umimayanthus* Montenegro, Sinniger & Reimer, 2015, and *Zibrowius* Sinniger, Ocaña & Baco, 2013.

### Parazoanthus

Taxon classificationAnimaliaZoanthariaParazoanthidae

Haddon & Shackelton, 1891

Heterozoanthus Verrill, 1870: 371 [*nomen oblitum*].
Parazoanthus Haddon & Shackleton, 1891a: 653, 654 [*nomen protectum*].

#### Type species.

*Palythoa axinella* Schmidt, 1862, by original designation.

#### Gender.

Masculine.

#### Diagnosis.

Originally described as well-developed canal system in the mesoglea of the column, forming a ring sinus. Zoantharians often associated with sponges but not Hydrozoa, lacking skeletal secretion (Sinniger et al. 2010a). Examined species with endodermal sphincter muscle (=branchiform endodermal muscle [Swain et al. 2015]), encrustations reaching to endodermal surface of mesoglea (Swain and Swain 2014).

#### Remarks.

“The original morphological description of *Parazoanthus* mentions several characteristics such as diffuse endodermal sphincter, encircling sinus, endodermal canals, lacunae and cell-islets in the mesoglea, continuous ectoderm and bodywall incrusted with mineral particles, often with numerous sponge spicules present in the incrustations. As shown in Sinniger et al. (2005) and here, these morphological characteristics alone do not ascertain the monophyly of *Parazoanthus*. Morphological characteristics in zoanthids can often become artifactual due to both complications encountered in making thin cuttings of heavily sediment incrusted polyps, and in interpreting the results of such sections. In the past, the large majority of epizoic macrocnemic zoanthids were described as belonging to *Parazoanthus* despite clearly different ecologies in many cases.

Thus, the results of this study strongly suggest that only zoanthid species able to associate with sponges should remain in *Parazoanthus*, as the type species of this genus, *Palythoa axinellae* from the Mediterranean Sea, is regularly associated with demosponges.” (Sinniger et al. 2010a: 69).

There is a need for a new diagnosis of this genus-grouping. With the recent erection of *Umimayanthus* Montenegro, Sinniger and Reimer, 2015 and the resurrection of *Bergia* Duchassaing de Fonbressin and Michelotti, 1860 in Montenegro et al. (2015a), the genus *Parazoanthus* now consists only of the former phylogenetic grouping of *Parazoanthus* ‘clade C’ sensu Sinniger et al. (2010a) and is monophyletic (Montenegro et al. 2015a: 71). *Parazoanthus* can be distinguished from *Bergia* and *Umimayanthus* by 16S-rDNA sequences, lacking the unique 60 bp deletion of *Bergia* and the unique insertion and deletion of *Umimayanthus*. Thus, the molecular characters described in Sinniger et al. (2008, 2013) appear to be efficient in identifying to genus level and could be used as diagnostic characters.

### Antipathozoanthus

Taxon classificationAnimaliaZoanthariaParazoanthidae

Sinniger, Reimer & Pawlowski, 2010

Antipathozoanthus Sinniger, Reimer & Pawlowski, 2010: 61.

#### Type species.

*Gerardia macaronesicus* Ocaña & Brito, 2003, by original designation.

#### Gender.

Masculine.

#### Diagnosis.

Sinniger et al. (2010a: 63) originally diagnosed this genus as a group that “grows exclusively on antipatharians” and lacking skeletal secretion.

#### Remarks.

No mesogleal canals or sinus, encrustation to outer mesoglea (Swain and Swain 2014), examined species with either branchiform endodermal or endo-meso transitional sphincter muscle (Swain et al. 2015).

Sinniger et al. (2010a: 63) discussed that “[t]he type species *Antipathozoanthus macaronesicus* was originally included in the description of Savalia (Gerardia) macaronesica (Ocaña and Brito 2003), and later the description was amended and the authors suggested the possible placement of this species in a separate genus (Ocaña et al. 2007). The species name was accorded to the genus gender. Skeletal secretion (similar to *Savalia* spp.) was advanced by Ocaña and Brito (2003) as occurring in *Antipathozoanthus macaronesicus*, and this remains to be studied in detail in order to assess whether this is an isolated characteristic or if it is taxonomically informative at genus level.”

It also appears at least one member of this genus can be found on gorgonian octocorals (Bo et al. 2012).

### Bergia

Taxon classificationAnimaliaZoanthariaParazoanthidae

Duchassaing de Fonbressin & Michelotti, 1860

Bergia Duchassaing de Fonbressin & Michelotti, 1860: 54.

#### Type species.

*Bergia catenularis* Duchassaing de Fonbressin & Michelotti, 1860, by subsequent designation by Duerden (1903: 496).

#### Gender.

Feminine.

#### Diagnosis.

“… can be distinguished from all other zoantharians including *Parazoanthus* spp., *Umimayanthus* spp. and *Epizoanthus* spp. by a unique deletion of 60 bp (from position 133 to 192 in our alignment) and several consecutive base substitutions in the 16S-rDNA region. These characters clearly separate this genus from all other genera inside the family Parazoanthidae, as well as from the genus *Epizoanthus*” (Montenegro et al. 2015a: 68).

#### Remarks.

Long considered to be a junior subjective synonym of *Parazoanthus* Haddon & Shackelton, 1891, recent molecular and morphological work by Montenegro et al. (2015a) have shown that the type species, *Bergia catenularis* Duchassaing de Fonbressin & Michelotti, 1860, represents a generic-level monophyly and resurrected the genus-group name *Bergia* Duchassaing de Fonbressin & Michelotti, 1860, for this grouping.

This genus-grouping currently contains three species all found in the Atlantic Ocean, although there is evidence of undescribed species in the Pacific Ocean (Montenegro et al. 2015a: 68).

Examined species in this genus-grouping have either branchiform endodermal or simplified mesogleal sphincter muscles (Swain et al. 2015).

### Bullagummizoanthus

Taxon classificationAnimaliaZoanthariaParazoanthidae

Sinniger, Ocaña & Baco, 2013

Bullagummizoanthus Sinniger, Ocaña & Baco, 2013: [9].

#### Type species.

*Bullagummizoathus emilyacadiaarum* Sinniger, Ocaña & Baco, 2013, by original designation.

#### Gender.

Masculine.

#### Diagnosis.

Characteristic insertion/deletion pattern in the 16S V5 region sensu Sinniger et al. (2005) (Sinniger et al. 2013).

#### Remarks.

Monospecific deep-sea genus, appears to be specifically epibiotic on paragorgiid octocorals, and described from and only reported from the Hawaiian Archipelago, although likely present throughout the Pacific (Sinniger et al. 2013).

### Corallizoanthus

Taxon classificationAnimaliaZoanthariaParazoanthidae

Reimer, in Reimer, Nonaka, Sinniger & Iwase, 2008

Corallizoanthus Reimer, in Reimer, Nonaka, Sinniger & Iwase, 2008: 940.

#### Type species.

*Corallizoanthus tsukaharai* Reimer, in Reimer, Nonaka, Sinniger & Iwase, 2008, by monotypy.

#### Gender.

Masculine.

#### Diagnosis.

Characterised by its association with living precious corals (Alcyonacea: Coralliidae), and unlike the gorgonian-associated genus *Savalia*, does not secrete its own scleroproteinous axis. Additionally, polyps are primarily but not always unitary (solitary; non-colonial) (see Reimer et al. 2008a: 940). Encrustations to center of mesoglea, sphincter muscle is cyclically transitional (Swain and Swain 2014, Swain et al. 2015).

#### Remarks.

A monospecific genus thus far only reported from the Ryukyu Islands, Japan.

### Hurlizoanthus

Taxon classificationAnimaliaZoanthariaParazoanthidae

Sinniger, Ocaña & Baco, 2013

Hurlizoanthus Sinniger, Ocaña & Baco, 2013: [7].

#### Type species.

*Hurlizoanthus parrishi* Sinniger, Ocaña and Baco, 2013, by original designation.

#### Gender.

Masculine.

#### Diagnosis.

Macrocnemic genus associated with primnoids. Characteristic insertion/deletion pattern in the 16S V5 region sensu Sinniger et al. (2005) (Sinniger et al. 2013).

#### Remarks.

Currently this deep-sea genus includes only one species, known from a few locations in the Hawaiian Archipelago (Sinniger et al. 2013).

### Isozoanthus

Taxon classificationAnimaliaZoanthariaParazoanthidae

Carlgren, in Chun, 1903

Polythoa (Taeniothoa) Andres, 1883a: 521, 532 [*nomen oblitum*].
Isozoanthus Carlgren, in Chun, 1903: 520 [*nomen protectum*].

#### Type species.

*Isozoanthus giganteus* Carlgren, in Chun, 1903, by monotypy (Articles 68.3, 68.3.1, ICZN, 1999: 71).

#### Gender.

Masculine.

#### Diagnosis.

Macrocnemic zoanthids with a marginal endodermal sphincter muscle (=cteniform endodermal [Swain et al. 2015]) and inconspicouous mesogleal ring-sinus.

#### Remarks.

The genus *Isozoanthus* was first made available in Carlgren (in Chun 1903: 520). The type species by monotypy is *Isozoanthus giganteus* (first very briefly diagnosed and figured by Carlgren (in Chun 1903: 520, unnumbered fig.). Carlgren’s (in Nordgaard 1905: 159) use of “Isozoanthus (Epizoanthus) arborescens” and subsequent designation (i.e. Carlgren 1913: 39) of *Epizoanthus arborescens* (Danielssen, 1890) as type species are thus invalid (Article 67.2, ICZN 1999: 66, 67) (see also Williams 2000: 195).

The status of this genus is currently very confused. With the utility of the characters of the sphincter muscle in zoantharians in question (see Swain 2010, Sinniger et al. 2010a), it is clear that more research is needed to clarify the diagnosis and taxonomic position of *Isozoanthus*. The taxonomy is further complicated by the recently described species (the hydroid-associated *Hydrozoanthus antumbrosus* (Swain, 2009) originally described within *Isozoanthus*), and the octocoral-associated *Isozoanthus primonodius* Carreiro-Silva, Braga-Henriques, Sampaio, de Matos, Porteiro & Ocaña, 2010) clearly belong to other genera based on ecology and morphology. Furthermore, only limited molecular data is available for the type species *Isozoanthus giganteus*. Data in Swain (2010) indicate that *Isozoanthus giganteus* is highly divergent from both the well-researched species of *Isozoanthus*—*Isozoanthus sulcatus* (Gosse, 1859)—and all known zoantharians. Recent work by Fujii and Reimer (2013) shows that *Isozoanthus sulcatus* is likely within the Nanazoanthidae Fujii & Reimer, 2013. However, as additional information on *Isozoanthus giganteus* is lacking, the diagnosis is retained with the caveat that any assignment of species to this genus should include: 1) phylogenetic confirmation of a close relationship with *Isozoanthus giganteus*; and 2) the elimination of any possibility that the species in question does not belong to another parazoanthid genera based on morphology, ecological associations, and/or habitat.

### Kauluzoanthus

Taxon classificationAnimaliaZoanthariaParazoanthidae

Sinniger, Ocaña & Baco, 2013

Kauluzoanthus Sinniger, Ocaña & Baco, 2013: [8].

#### Type species.

*Kauluzoanthus kerbyi* Sinniger, Ocaña & Baco, 2013, by original designation.

#### Gender.

Masculine.

#### Diagnosis.

Polyps do not contract when fixed. Characteristic insertion/deletion pattern in the 16S V5 region sensu Sinniger et al. (2005) (Sinniger et al. 2013).

#### Remarks.

Currently this genus comprises only one species, which was reported as parasitic on *Kulamanana haumeaae*.

### Kulamanamana

Taxon classificationAnimaliaZoanthariaParazoanthidae

Sinniger, Ocaña & Baco, 2013

Kulamanamana Sinniger, Ocaña & Baco, 2013: 4.

#### Type species.

*Kulamanamana haumaeaae* Sinniger, Ocaña & Baco, 2013, by original designation.

#### Gender.

Feminine.

#### Diagnosis.

Macrocnemic genus associated with octocorals and secreting a golden to dark brown scleroproteic skeleton. Ectoderm absent of mineral particles, with well-developed coenenchyme completely covering the host. Characteristic insertion/deletion pattern in the 16S V5 region sensu Sinniger et al. (2005) (Sinniger et al. 2013).

#### Remarks.

The type species has been reported to live primarily on isidid corals (bamboo corals) (Sinniger et al. 2013: 6). Reported from the Hawaiian Archipelago, Line and Jarvis Islands, Palmyra Atoll, Kingman Reef, all in the Pacific (Sinniger et al. 2013).

### Mesozoanthus

Taxon classificationAnimaliaZoanthariaParazoanthidae

Sinniger & Häussermann, 2009

Mesozoanthus Sinniger & Häussermann, 2009: 31, 32.

#### Type species.

*Mesozoanthus fossii* Sinniger & Häussermann, 2009, by original designation and monotypy.

#### Gender.

Masculine.

#### Diagnosis.

“Macrocnemic with *Parazoanthus*-like growth-form. Well-developed polyps with long and pointed tentacles; polyps form clusters linked by a basal coenenchyme. DNA sequences significantly differ from those in other genera...” and “In contrast to *Parazoanthus*, members of *Mesozoanthus* usually occur in small patches and are not known to colonise demosponges. No symbioses with *Symbiodinium* zooxanthellae.” (Sinniger and Häussermann 2009: 32).

#### Remarks.

Only two species of this genus are known, from temperate waters along the west coast of the Americas.

### Savalia

Taxon classificationAnimaliaZoanthariaParazoanthidae

Nardo, 1844

Savalia Nardo, 1844: 433, 434.
Savaglia Nardo, 1877: 674–676 [unjustified emendation].
Gerardia Lacaze-Duthiers, 1864a: 87.

#### Type species.

*Gorgonia savaglia* Bertolini, 1819, by monotypy (Articles 68.3, 68.3.1, ICZN 1999: 71).

#### Gender.

Masculine.

#### Diagnosis.

No mesogleal canal system in the column. Secreting a black or dark brown horny skeleton, azooxanthellate.

#### Remarks.

Examined species in this genus-grouping have branchiform endodermal or cyclical transitional sphincter muscle (Swain et al. 2015). Distinction from the other skeleton-secreting zoantharians such as *Kulamanamana* or potentially *Antipathozoanthus* can be made through habitat (shallow vs. deep-sea) and/or molecular signatures.

There has been historical and recent controversy over the correct name for this genus-group, and this is discussed in detail in Appendix 3.

### Umimayanthus

Taxon classificationAnimaliaZoanthariaParazoanthidae

Montenegro, Sinniger & Reimer, 2015

Umimayanthus Montenegro, Sinniger & Reimer, 2015: 76.

#### Type species.

*Umimayanthus chanpuru* Montenegro, Sinniger & Reimer, 2015, by original designation.

#### Gender.

Masculine.

#### Diagnosis.

“…can be distinguished from all zoantharians including *Parazoanthus* spp. by a highly conservative and unique insertion of 9 bp in length (from position 556 to 564 in alignment) and one deletion of 14 bp long (from position 574 to 587) in the mt 16S-rDNA region” (Montenegro et al. 2015b: 76).

#### Remarks.

Specimens examined from this genus-grouping have a branchiform endodermal sphincter muscle (Swain et al. 2015).

“…exclusively associated with sponges, usually encrusting and cushion sponges, occasionally with massive sponges. Polyps generally scattered over the sponge surface, but can form defined stoloniferous chains in lines, or form groups of two to three connected polyps. Polyps may be solitary or connected to each other by a stolon through a thin but clearly visible coenenchyme either over or under the sponge surface. Polyps with sand particles and detritus incrusted in column. Tentacles equal or longer than the expanded oral disc diameter.” (Montenegro et al. 2015b: 76).

This genus-group currently includes four described species; three in the Indo-Pacific and one in the Atlantic (Montenegro et al. 2015b).

### Zibrowius

Taxon classificationAnimaliaZoanthariaParazoanthidae

Sinniger, Ocaña & Baco, 2013

Zibrowius Sinniger, Ocaña & Baco, 2013: [7].

#### Type species.

*Zibrowius ammophilus* Sinniger, Ocaña & Baco, 2013, by original designation.

#### Gender.

Masculine.

#### Diagnosis.

“Sand incrusted, arborescent fan shaped colonies, golden skeleton, well developed coenenchyme completely covering the host, can be confused with *Kulamanamana*, but easily distinguished by the presence of sand incrustation in the ectoderm, characteristic insertion/deletion pattern in the 16S V5 region” sensu Sinniger et al. [2005] (Sinniger et al. 2013: 7)

#### Remarks.

Until now, only reported from the Cross Seamount in the central Pacific Ocean.

### Suborder *incerta sedis*

**Remarks.** Two families are currently not assigned to any suborder.

#### Abyssoanthidae

Taxon classificationAnimaliaZoanthariaAbyssoanthidae

Reimer & Fujiwara, in Reimer, Sinniger, Fujiwara, Hirano & Maruyama, 2007

Abyssoanthidae Reimer & Fujiwara, in Reimer, Sinniger, Fujiwara, Hirano & Maruyama, 2007: 258.

##### Type genus.

*Abyssoanthus* Reimer & Fujiwara, in Reimer, Sinniger, Fujiwara, Hirano & Maruyama, 2007.

##### Diagnosis.

“Sand/detritus/sediment-encrusted Zoantharia with unitary (noncolonial) free-living polyps, attached to hard substrates at abyssal (non-continental shelf deep-sea) depths surrounding methane cold seeps or other chemosynthetic ecosystems.” Reimer and Sininger (2010: 454).

##### Remarks.

A monogeneric family. Molecular data places this family in an unresolved position distant from the Brachycnemina and Macrocnemina.

#### Abyssoanthus

Taxon classificationAnimaliaZoanthariaAbyssoanthidae

Reimer & Fujiwara, in Reimer, Sinniger, Fujiwara, Hirano & Maruyama, 2007

Abyssoanthus Reimer & Fujiwara, in Reimer, Sinniger, Fujiwara, Hirano & Maruyama, 2007: 258.

##### Type species.

*Abyssoanthus nankaiensis* Reimer & Fujiwara, in Reimer, Sinniger, Fujiwara, Hirano & Maruyama, 2007, by original designation and monotypy.

##### Gender.

Masculine.

##### Diagnosis.

As for family above.

##### Remarks.

Two species known, both from waters around Japan. There may be additional species from the Mediterranean, in non-chemosynthetic environments (Sinniger et al. 2010b).

#### Family *incerta sedis*

##### Stephanidium

Taxon classificationAnimaliaZoanthariaAbyssoanthidae

Hertwig, 1888

Stephanidium Hertwig, 1888: 52.

###### Type species.

*Stephanidium schulzii* Hertwig, 1888, by monotypy.

###### Gender.

Neuter.

###### Diagnosis.

Very small (diameter 1.5–2.2 mm, height 1 mm), unitary polyps, with microcnemes and macrocnemes, although their arrangement could not be clearly seen. Mesenteriel insertions make body wall to have a furrowed appearance, with spherical evaginations on the body wall above the area where the sphincter muscle is present. Twenty-six mesenteries.

###### Remarks.

From the original description, possibly a species of zoantharian, but type material needs to be located and examined. We make no decision as to the validity of this genus and species in the event that the identification of this genus and species requires a reversal of precedence Article 23.9 (ICZN 1999: 27, 28) with a later (but more widely-used name). Also, the genus-group name *Stephanidium* Hertwig, 1888, is preoccupied by *Stephanidium* Ehrenberg, 1839 (*incerta sedis*).

## Discussion

As can be seen from examining the nomenclatural and taxonomic history of the various supraspecific names in this paper, many taxa of the order Zoantharia have a confused history. However, over the past two decades, phylogenetic and detailed morphological examinations of zoantharians have resulted in a new understanding of the evolutionary relationships within the order (Sinniger et al. 2005, Reimer et al. 2007a, 2008a, Reimer and Fujii 2010, Sinniger et al. 2010a, 2013, Fujii and Reimer 2011, 2013; Swain et al. 2015). Combined with a recent effort to organize the nomenclature of zoantharians (Low and Reimer 2011a, b, 2012a, b), it can be said that much of the Zoantharia nomenclature is now stable, and generally reflects our current understanding of their evolutionary history.

However, as seen in this manuscript, there are still some nomenclatural issues that remain to be resolved. For example, the validity of the genera *Sphenopus* and *Acrozoanthus* still have to be thoroughly examined. Furthermore, it is clear from recent molecular phylogenetic results (Fujii and Reimer 2013) that the taxonomy and nomenclature of the suborders are in urgent need of a revision. Finally, at the ordinal level, it appears that Zoantharia is much closer to the Actiniaria (sea anemones) than has been previously thought (Fujii and Reimer 2013). As these two orders have been speculated to be basal cnidarians (Kayal et al. 2013), after clarification of their evolutionary history, subsequent nomenclatural amendment may be needed.

Additional work examining the utility of ecological and morphological traits of Zoantharia as diagnostic characters is needed to allow the linkage of current phylogenetic results with past literature (Swain et al. 2016). Despite, or perhaps due to its confused and challenging morphological taxonomy, the order Zoantharia is now among the most advanced hexacorallian order in terms of the use of molecular tools to clarify phylogenetic relationships between taxa at various taxonomic levels. As such, and despite potential and known differences in molecular evolution of other anthozoans (Stampar et al. 2014), Zoantharia is a potentially good model for clarifying the taxonomy of other hexacorallian orders and anthozoan groups.

## Supplementary Material

XML Treatment for Cnidaria

XML Treatment for Anthozoa

XML Treatment for Hexacorallia

XML Treatment for Zoantharia

XML Treatment for Brachycnemina

XML Treatment for Neozoanthidae

XML Treatment for Neozoanthus

XML Treatment for Sphenopidae

XML Treatment for Sphenopus

XML Treatment for Palythoa

XML Treatment for Zoanthidae

XML Treatment for Zoanthus

XML Treatment for Acrozoanthus

XML Treatment for Isaurus

XML Treatment for Macrocnemina

XML Treatment for Epizoanthidae

XML Treatment for Epizoanthus

XML Treatment for Palaeozoanthus

XML Treatment for Thoracactis

XML Treatment for Hydrozoanthidae

XML Treatment for Hydrozoanthus

XML Treatment for Terrazoanthus

XML Treatment for Microzoanthidae

XML Treatment for Microzoanthus

XML Treatment for Nanozoanthidae

XML Treatment for Nanozoanthus

XML Treatment for Parazoanthidae

XML Treatment for Parazoanthus

XML Treatment for Antipathozoanthus

XML Treatment for Bergia

XML Treatment for Bullagummizoanthus

XML Treatment for Corallizoanthus

XML Treatment for Hurlizoanthus

XML Treatment for Isozoanthus

XML Treatment for Kauluzoanthus

XML Treatment for Kulamanamana

XML Treatment for Mesozoanthus

XML Treatment for Savalia

XML Treatment for Umimayanthus

XML Treatment for Zibrowius

XML Treatment for Abyssoanthidae

XML Treatment for Abyssoanthus

XML Treatment for Stephanidium

## Appendix 1

Nomenclator of family-group names in the Zoantharia Rafinesque, 1815

Names in **bold** and designated with an asterisk (*) are considered to be valid family-group names. Also refer to the genus-group names in the next section. Names marked with a † are invalid synonyms, or incorrect spellings (if further denoted by a “[*sic*]”). Additional names without either designation have been confused with the order Zoantharia at one time or another but have since been removed from this group.

***Abyssoanthidae** Reimer & Fujiwara, in Reimer, Sinniger, Fujiwara, Hirano & Maruyama, 2007: 258. Type genus *Abyssoanthus* Reimer & Fujiwara, in Reimer, Sinniger, Fujiwara, Hirano & Maruyama, 2007.

Bergiidae Verrill, 1865. See Bergiidae Verrill, 1865.

†Bergiidae [*sic*]. Incorrect subsequent spelling of Bergiidae Verrill, 1865 (e.g. Verrill 1869 [in 1868–1871]: 494; Duerden 1903: 495).

†Bergiidae Verrill, 1865a: 147. Type genus *Bergia* Duchassaing de Fonbressin & Michelotti, 1860. This family-group name was first established as “Bergiidae” (an incorrect original spelling. *Bergia* Duchassaing de Fonbressin & Michelotti, 1860, was previously considered to be a junior subjective synonym of *Parazoanthus* Haddon & Shackleton, 1891, but has since been revalidated (see Montenegro et al. 2015a: 63–71). Bergiidae Verrill, 1865, and Parazoanthidae Delage & Hérouard, 1901, nevertheless remain subjective synonyms. Low and Reimer (2011a: 64, 65) enacted Article 23.9 (ICZN 1999: 27, 28) to reverse precedence between these family-group names, thereby making Bergiidae Verrill, 1865, a *nomen oblitum*, and Parazoanthidae Delage & Hérouard, 1901, a *nomen protectum*. Low and Reimer (2011a: 64, 65) incorrectly attributed the name Bergiidae to Verrill, 1869 (in 1868–1871), but the name should be attributed to Verrill (1865). The findings and action of Low and Reimer (2011: 64, 65) remain valid.

***Epizoanthidae** Delage & Hérouard, 1901: 664. Type genus *Epizoanthus* Gray, 1867. First described as the subfamily “Epizoanthinae”. See also Mardoellidae Danielssen, 1890.

Gerardiidae Verrill, 1865. See Gerardiidae Verrill, 1865a.

†Gerardiidae [*sic*]. Incorrect subsequent spelling of Gerardiidae Verrill, 1865 (e.g., Verrill 1869 [in 1868–1871]: 499; Duerden 1903: 495).

†Gerardiidae Verrill, 1865a: 1484. Type genus *Gerardia* Lacaze-Duthiers, 1864. This family-group name was first established as “Gerardiidae” (an incorrect original spelling). A subjective synonym of Parazoanthidae Delage & Hérouard, 1901. This family group name is a senior subjective synonym of Parazoanthidae Delage & Hérouard, 1901, and an application to the ICZN in accordance with Article 23.9.2 (ICZN 1999: 28, 29) is in preparation to suppress this name in favour of the more universally-accepted and -used name Parazoanthidae Delage & Hérouard, 1901. Also see discussion in Appendix 3.

†Heterozoanthidae Pax & Müller, 1956: 2. Type genus *Heterozoanthus* Verrill, 1870. The type genus is an objective synonym of *Parazoanthus* Haddon and Shackleton, 1891, making Heterozoanthidae Pax & Müller, 1956, a junior objective synonym of Parazoanthidae Delage and Hérouard, 1901 (Article 61.3.2 of the Code, ICZN 1999: 64) (see Low and Reimer 2012a: 83, 84).

***Hydrozoanthidae** Sinniger, Reimer & Pawlowski, 2010: 60. Type genus *Hydrozoanthus* Sinniger, Reimer & Pawlowski, 2010.

†Mardoellidae Danielssen, 1890: 116, 117. Type genus *Mardoell* Danielssen, 1890. The type genus is a subjective synonym of *Epizoanthus* Gray, 1867 (see Low and Reimer 2011b: 84, 85; Lwowsky 1913: 603, 604). Mardoellidae Danielssen, 1890, is therefore a subjective synonym of Epizoanthidae Delage & Hérouard, 1901. Low and Reimer (2011b: 84) enacted Article 23.9 (ICZN 1999: 27, 28) to reverse precedence between these family-group names, thereby making Mardoellidae Danielssen, 1890, a *nomen oblitum*, and Epizoanthidae Delage and Hérouard, 1901, a *nomen protectum*.

***Microzoanthidae** Fujii & Reimer, 2011: 420, 421. Type genus *Microzoanthus* Fujii & Reimer, 2011.

***Nanozoanthidae** Fujii & Reimer, 2013: 512. Type genus *Nanozoanthus* Fujii & Reimer, 2013.

***Neozoanthidae** Herberts, 1972: 137. Type genus *Neozoanthus* Herberts, 1972.

Orinidae Verrill, 1869. See Oriniidae Verrill, 1869.

Oriniidae Verrill, 1869 (in 1868–1871): 494. Type genus *Orinia* Duchassaing de Fonbressin & Michelotti, 1860. This family-group name was first established as “Orinidae” (an incorrect original spelling). The type genus *Orinia* Duchassaing de Fonbressin & Michelotti, 1860, is a junior subjective synonym of *Rhodactis* Milne Edwards & Haime, 1851, which is currently assigned to the family Discosomidae Verrill, 1869 [Coralliomorpha] (see Fautin 2016: 25, 28, 38). Also see remarks under *Orinia* Duchassaing de Fonbressin & Michelotti, 1860, in Appendix 2.

Polythoae Duchassaing de Fonbressin & Michelotti, 1860. See Palythoidae Duchassaing de Fonbressin & Michelotti, 1860.

Palythoidae Duchassaing de Fonbressin & Michelotti, 1860: 37. Type genus *Palythoa* Lamouroux, 1816 (spelled as “*Polythoa*”). This family-group name was first established as “Polythoae” (based on “*Polythoa*”, an incorrect subsequent spelling of *Palythoa* Lamouroux, 1816, see Appendix 3). A subjective synonym of Sphenopidae Hertwig, 1882, as the type species *Sphenopus* Steenstrup, 1856, and *Palythoa* Lamouroux, 1816, as assigned to the same family (see Appendix 3). Precedence between Palythoidae Duchassaing de Fonbressin & Michelotti, 1860, and Sphenopidae Hertwig, 1882, cannot be reversed and an application to the ICZN in accordance with Article 23.9.2 (ICZN 1999: 28, 29) is in preparation to suppress the former name in favour of the latter name that is more widely accepted and used. Also see discussion in Appendix 3.

***Parazoanthidae** Delage & Hérouard, 1901: 665. Type genus *Parazoanthus* Haddon & Shackleton, 1891. See also the synonyms Bergiidae Verrill, 1865, Gerardiidae Verrill, 1865, Heterozoanthidae Pax & Müller, 1956, Savagliidae Brook, 1889, and Savaliidae Nardo, 1844. Also see discussion in Appendix 3.

†Savagliidae Brook, 1889: 51, 74, 79. Type genus *Savaglia* Nardo, 1877. A subjective synonym of Parazoanthidae Delage & Hérouard, 1901. This family group name is a senior subjective synonym of Parazoanthidae Delage & Hérouard, 1901, and an application to the ICZN in accordance with Article 23.9.2 (ICZN 1999: 28, 29) is in preparation to suppress this name in favour of the more widely accepted and used name Parazoanthidae Delage & Hérouard, 1901. Also see discussion in Appendix 3.

†Savaliidae Nardo, 1844: 433. Type genus *Savalia* Nardo, 1844. This family-group name was first established at the rank of subfamily as “Savalini” (an incorrect original spelling). A subjective synonym of Parazoanthidae Delage and Hérouard, 1901. This family group name is a senior subjective synonym of Parazoanthidae Delage & Hérouard, 1901, and an application to the ICZN in accordance with Article 23.9.2 (ICZN 1999: 28, 29) is in preparation to suppress this name in favour of the more universally-accepted and -used name Parazoanthidae Delage & Hérouard, 1901. Also see discussion in Appendix 3.

†Savaliidae Poche, 1914: 104. Type genus *Savalia* Nardo, 1844. Proposed as a replacement name for Savagliidae Brook, 1889. A junior objective synonym of Savaliidae Nardo, 1844, and Savagliidae Brook, 1889.

Savalini Nardo, 1844. See Savaliidae Nardo, 1844.

***Sphenopidae** Hertwig, 1882: 120. Type genus *Sphenopus* Steenstrup, 1856.

Zoanthia Rafinesque, 1815. See Zoanthidae Rafinesque, 1815.

***Zoanthidae** Rafinesque, 1815: 155. Type genus *Zoanthus* Lamarck, 1801. First established at the rank of subfamily as “Zoanthia” (an incorrect original spelling). Authorship of the family-group name Zoanthidae is conventionally attributed to Gray (1832: 95; 1840: 72), however Rafinesque’s (1815) indication has priority (see Low and Reimer 2012a: 85).

## Appendix 2.

Nomenclator of genus-group names in the Zoantharia Rafinesque, 1815

Names in **bold** and designated with an asterisk (*) are considered to be valid genus-group names. In accordance with Article 67.2 of the Code (ICZN 1999: 66, 67), all type species designations made herein are from among the “originally included nominal species”. Names marked with a † are invalid synonyms, or incorrect spellings (if further denoted by a “[*sic*]”). Additional names without either designation have been confused with the order Zoantharia at one time or another but have since been removed from this group. The identity of two possible zoantharian genus-group name-*Stephanidium* Hertwig, 1888, and *Triga* Gray, 1867-will need to be resolved.

****Abyssoanthus*** Reimer & Fujiwara, in Reimer, Sinniger, Fujiwara, Hirano & Maruyama, 2007: 258. Type species *Abyssoanthus nankaiensis* Reimer & Fujiwara, in Reimer, Sinniger, Fujiwara, Hirano & Maruyama, 2007, by original designation; gender masculine.

****Acrozoanthus*** Saville-Kent, 1893: 153, 154, unnumbered fig. Type species *Acrozoanthus australiae* Saville-Kent, 1893, by monotypy; gender masculine.

†*Actimastus* Rafinesque, 1818: 271. Replacement name for *Mammillifera* Le Sueur, 1817. Type species *Mammillifera auricula* Le Sueur, 1817, by subsequent designation by Haddon and Shackleton (1891a: 626) (for *Mammillifera* Le Sueur, 1817); gender masculine. Unnecessary replacement name for and junior objective synonym of *Mammilifera* Le Sueur, 1817. Both *Actimastus* Rafinesque, 1818, and *Mammilifera* Le Sueur, 1817, are junior subjective synonyms of *Zoanthus* Lamarck, 1801, as the type species of both genus-group names is now considered to be a species of *Zoanthus* Lamarck, 1801 (see Appendix 3).

*Anthozoanthus* Carter, 1870: 449. Type species *Anthozoanthus parasiticus*, by monotypy; gender masculine. Not a zoantharian (see Carter 1870: 449). This name appears to be Deshayes manuscript name that was published by Carter (1870).

†*Anthozoon* Gistel, 1848: 181. Unnecessary replacement name and junior objective synonym of *Zoanthus* Lamarck, 1801; gender neuter.

†*Antinedia* Duchassaing de Fonbressin & Michelotti, 1864: 42 (also 1866: 136). Type species *Zoanthus tuberculatus* Duchassaing de Fonbressin, 1850, by monotypy; gender feminine. The type species *Zoanthus tuberculatus* Duchassaing de Fonbressin, 1850, is a junior subjective synonym and junior secondary homonym of *Isaurus tuberculatus* Gray, 1828. A subjective junior synonym of *Isaurus* Gray, 1828 (see Muirhead and Ryland 1985: 325).

†*Actinorhiza* Agassiz, 1846: 7. Unjustified emendation and junior objective synonym of *Actinorhyza* Blainville, 1830. See Appendix 3.

†*Actinorhyza* [*sic*]. Incorrect subsequent spelling of *Actinorhyza* Blainville, 1830, by Blainville (1834: 329). See Appendix 3.

† *Actinorhyza* Blainville, 1830: 295. Type species *Actinia sociata* Ellis, 1768 (see Appendix 3); gender feminine. An unnecessary replacement name for, and junior objective synonym of *Zoanthus* Lamarck, 1801. See also the incorrect subsequent spellings *Actinorhysa* Blainville, 1834, and *Actinorrhyza* Ehrenberg, 1834, and the unjustified emendation *Actinorhiza* Agassiz, 1844. See additional discussion in Appendix 3.

†*Actinorhyza* [*sic*]. Incorrect subsequent spelling of *Actinorhyza* Blainville, 1830, by Ehrenberg (1834a: 269). See Appendix 3.

*Actinocereus* Blainville, 1830: 294. No type species designated (see Fautin et al. 2012: 20, 21); gender masculine. Placed in synonymy (in part) of *Hughea* Lamouroux, 1821, by Andres (1881: 336). Currently a genus-group name in the Actiniaria (see Fautin et al. 2012: 20–22; Fautin 2016: 54). *Actinocereus* Blainville, 1830, is now considered a synonym of *Cereus* Ilmoni, 1830, and the International Commission for Zoological Nomenclature was petitioned Fautin et al. (2012: 22) to suppress the former name in favour of the latter, and subsequenty ruled as such (ICZN 2014: 136, 137).

****Antipathozoanthus*** Sinniger, Reimer & Pawlowski, 2010: 61. Type species *Gerardia macaronesicus* Ocaña & Brito, 2003, by original designation; gender masculine.

*Axinella* O. Schmidt, 1862: 60. Type species *Axinella polypoides* O. Schmidt, 1862 (see Gazave et al. 2010). Not a zoantharian (see Gazave et al. 2010).

****Bergia*** Duchassaing de Fonbressin & Michelotti, 1860: 54. Type species *Bergia catenularis* Duchassaing de Fonbressin & Michelotti, 1860, by subsequent designation by Duerden (1903: 496); gender feminine. The type species was formerly assigned to the genus *Parazoanthus* Haddon & Shackleton, 1891a (see Duerden 1903: 496, Low and Reimer 2011a: 64), making *Bergia* Duchassaing de Fonbressin & Michelotti, 1860, a subjective synonym of *Parazoanthus* Haddon & Shackleton, 1891a. Low and Reimer (2011a) enacted Article 23.9 (ICZN 1999: 27, 28) to reverse precedence between these genus-group names, thereby making *Bergia* Duchassaing de Fonbressin & Michelotti, 1860, a *nomen oblitum*, and *Parazoanthus* Haddon & Shackleton, 1891a, a *nomen protectum*. Recent molecular and morphological work by Montenegro et al. (2015a: 63–71) have shown that the type species, *Bergia catenularis* Duchassaing de Fonbressin & Michelotti, 1860, represents a generic-level monophyly and resurrected the genus-group name *Bergia* Duchassaing de Fonbressin & Michelotti, 1860, for this grouping.

****Bullagummizoanthus*** Sinniger, Ocaña & Baco, 2013: 9. Type species *Bullagummizoanthus emilyacadiaarum* Sinniger, Ocaña & Baco, 2013, by original designation; gender masculine.

†*Carolia* Gray, 1867: 239. Type species *Zoanthus couchii* Johnston, in Couch, 1844, by monotypy; gender feminine. The type species is now assigned to the genus *Epizoanthus* Gray, 1867 (see Haddon and Shackleton 1891a: 644, 645), making *Carolia* Gray, 1867, a subjective synonym of *Epizoanthus* Gray, 1867. The name *Carolia* Gray, 1867, is an invalid junior homonym of *Carolia* Cantraine, 1838 (Mollusca), and the *Epizoanthus* Gray, 1867, is the valid name for this genus-group. See also *Epizoanthus* Gray, 1867.

†*Cavolinia* Schweigger, 1819: 99. No type species designated, for originally included species, see Appendix 3; gender feminine. *Cavolinia* Schweigger, 1819, is a junior homonym of *Cavolinia* Abildgaard, 1791, and is not a valid name (see Appendix 3). See also the unnecessary replacement name *Cynicus* Gistel, 1848.

****Corallizoanthus*** Reimer, in Reimer, Nonaka, Sinniger & Iwase, 2008: 940. Type species *Corallizoanthus tsukaharai* Reimer, in Reimer, Nonaka, Sinniger & Iwase, 2008, by original designation; gender masculine.

†*Corticanthus* Andres, 1883a: 538, 541. Type species *Epizoanthus paguriphilus* Verrill, 1882, herein designated (see Appendix 3); gender masculine. First proposed as a subgenus of *Zoanthus* Lamarck, 1801. The type species is now assigned to the genus *Epizoanthus* Gray, 1867 (see Appendix 3), making Zoanthus (Corticanthus) Andres, 1883, a junior subjective synonym of *Epizoanthus* Gray, 1867.

†*Corticifera* Le Sueur, 1817: 178, 179. Type species *Corticifera glareola* Le Sueur, 1817, by subsequent designation by Haddon and Shackleton (1891b: 692); gender feminine. The type species is now assigned to the genus *Palythoa* Lamouroux, 1816 (see Appendix 3), making *Corticifera* Le Sueur, 1817, a junior subjective synonym of *Palythoa* Lamouroux, 1816.

†*Corticithoa* Andres, 1883a: 521, 535–538. Type species *Alyconium tuberculosum* Esper, 1805, herein designated (see Appendix 3); gender feminine. First proposed as a subgenus of *Palythoa* Lamouroux, 1816. The type species, *Alcyonium tuberculosum*, is now assigned to the genus *Palythoa* Lamouroux, 1816 (see Appendix 3), making Palythoa (Corticithoa) Andres, 1883, a junior subjective synonym of *Palythoa* Lamouroux, 1816.

†*Cortificera*. Incorrect spelling of *Corticifera* Le Sueur, 1817, by Brandt (1835: 208).

†*Cynicus* Gistel, 1848: viii. Unnecessary replacement name for, and junior objective synonym of, *Cavolinia* Schweigger, 1819; gender masculine.

*Edwardsia* de Quatrefages, 1841: 427. Type species *Edwardsia beautempsii* de Quatrefages, 1842, by subsequent designation by Delphy (1938) (see Fautin 2016: 82); gender feminine. Not a zoantharian (see Fautin et al. 2007: 201, Williams 1981a: 326; Fautin 2016: 82, 83).

†*Endeithoa* Andres, 1883a: 521, 531. Type species *Zoanthus norvegicus* Koren and Danielssen, 1877, herein designated (see Appendix 3); gender feminine. First proposed as a subgenus of *Palythoa* Lamouroux, 1816. The type species is now assigned to the genus *Epizoanthus* Gray, 1867 (see Appendix 3), making Palythoa (Endeithoa) Andres, 1883, a junior subjective synonym of *Epizoanthus* Gray, 1867.

*Epiactis* Verrill, 1869 (in 1868–1871): 492. Type species *Epiactis prolifera* Verrill, 1869, by monotypy; gender feminine. Not a zoantharian (Fautin et al. 2007: 202; Fautin 2016: 85).

****Epizoanthus*** Gray, 1867: 237. Type species *Dysidea papillosa* Johnston, 1842, by monotypy; gender masculine. Placed on *Official List* and has priority over *Sidisia* Gray, 1858 (see Opinion 1689, ICZN 1992: 236). The subsequent designation of *Mammillifera incrustatus* Düben & Koren, 1847, as the type species of *Epizoanthus* Gray, 1867, by Haddon and Shackleton (1891a: 627) is not valid as this species was not amongst the species originally included in the description of *Epizoanthus* Gray, 1867, and does not qualify for selection as the type species (see Ryland and Muirhead 1991: 19, 20; and Article 69.2 of the Code, ICZN 1999: 72, 73). See also the synonyms *Carolia* Gray, 1867, *Lirrevia* Delphy, 1939: 270, *Mardoell* Danielssen, 1890, *Marodellia* Danielssen, 1890, Palythoa (Endeithoa) Andres, 1883, *Sidisia* Gray, 1858, *Verrillia* Andres, 1883, and Zoanthus (Corticanthus) Andres, 1883.

†*Gemmaria* Duchassaing de Fonbressin & Michelotti, 1860: 55. Type species *Gemmaria rusei* Duchassaing de Fonbressin & Michelotti, 1860, by subsequent designation by Haddon and Shackleton (1891a: 626). The genus-group name *Gemmaria* Duchassaing de Fonbressin & Michelotti, 1860, is preoccupied by *Gemmaria* McCrady, 1859 (Hydrozoa). Herein considered to be a junior subjective synonym of *Palythoa* Lamouroux, 1816 (See Appendix 3). See also the replacement names *Haplotella* Stechow, 1919, and *Protopalythoa* Verrill, 1900.

†*Gemmithoa* Andres, 1883a: 521, 532, 533. Type species *Mammillifera brevis* Duchassaing de Fonbressin, 1850, by monotypy; gender feminine. First proposed as a subgenus of *Palythoa* Lamouroux, 1816. The type species is now assigned to the genus *Palythoa* Lamouroux, 1816 (see Appendix 3), making Palythoa (Gemmithoa) Andres, 1883, a junior subjective synonym of *Palythoa* Lamouroux, 1816.

†*Gerardia* Lacaze-Duthiers, 1864a: 87. Type species *Leoipathes lamarcki* Haime, 1849, by monotypy; gender feminine. A subjective junior synonym of *Savalia* Nardo, 1844 (see Appendix 3). This genus-group name was also described as new in Lacaze-Duthiers (1864c: 175, 176), and appeared as a translation in Lacaze-Duthiers (1864b: 242).

†*Gerardina* [sic]. Incorrect spelling of *Gerardia* Lacaze-Duthiers, 1864, by H. Schmidt (1972: 452).

†*Haplotella* Stechow, 1919: 853. Replacement name for *Gemmaria* Duchassaing de Fonbressin & Michelotti, 1860, preoccupied by *Gemmaria* McCrady, 1859 (Hydrozoa). Type species *Gemmaria rusei* Duchassaing de Fonbressin & Michelotti, 1860, by typification of the preoccupied name; gender feminine. A junior objective synonym of *Gemmaria* Duchassaing de Fonbressin & Michelotti, 1860, and a junior subjective synonym of *Palythoa* Lamouroux, 1816 (see Appendix 3). See also *Gemmaria* Duchassaing de Fonbressin & Michelotti, 1860, *Parapalythoa* Verrill, 1900, and *Protopalythoa* Verrill, 1900.

†*Heterozoanthus* Verrill, 1870: 371. Type species *Palythoa axinellae* O. Schmidt, 1862, by subsequent designation by Low and Reimer (2012a: 84); gender masculine. As *Heterozoanthus* Verrill, 1870, and *Parazoanthus* Haddon and Shackleton, 1891, have the same type species, they are objective synonyms. Low and Reimer (2012a: 84) reversed precedence thereby making *Heterozoanthus* Verrill, 1870, a *nomen oblitum*, and *Parazoanthus* Haddon & Shackleton, 1891, a *nomen protectum*.

*Hughea* [*sic*]. Incorrect spelling of *Hughea* Lamouroux, 1821, by Duchassaing de Fonbressin (1850: 9). Not a zoantharian (see *Hughea* Lamouroux, 1821).

*Hughea* Lamouroux, 1821: 89. Type species *Actinia calendula* Hughes, in Ellis & Solander, 1786, by monotypy; gender feminine. The type species, *Actinia calendula* was described by Ellis, in Ellis and Solander (1786: 7, 8, pl. 1, fig. 3) based on an unnamed cnidarian described and illustrated by Hughes (1750: 293, 294, pl. 24, fig. 1) that is clearly not a zoantharian (see also Ryland and Lancaster 2003: 411; Fautin 2016: 182). *Actinia calendula* Ellis, in Ellis & Solander, 1786, is currently considered to be assigned to the genus *Petalactis* Andres, 1883 (see Fautin 2016: 118), thereby making *Hughea* Lamouroux, 1821, a synonym of *Petalactis* Andres, 1883. Also see the unnecessary replacement name *Meto* Gistel, 1848.

*Hughea* [*sic*]. Incorrect spelling of *Hughea* Lamouroux, 1821, by Gistel (1848: 181). Not a zoantharian (see *Hughea* Lamouroux, 1821).

****Hurlizoanthus*** Sinniger, Ocaña & Baco, 2013: 7. Type species *Hurlizoanthus parrishi* Sinniger, Ocaña & Baco, 2013, by original designation; gender masculine.

****Hydrozoanthus*** Sinniger, Reimer & Pawlowski, 2010: 60. Type species *Parazoanthus tunicans* Duerden, 1900, by original designation; gender masculine.

*Iluanthos* Forbes, 1840: 184. Type species *Iluanthos scoticus* Forbes, 1840, by monotypy; gender masculine. Fautin (2016: 97) considers the correct original spelling of this genus-group name to be “Ilyanthus” following Article 33.3.1 of the Code (ICZN 1999: 43). Not a zoantharian (Fautin et al. 2007: 209; Fautin 2016: 97).

†*Isaurus* [*sic*]. Incorrect subsequent spelling of *Isaurus* Gray, 1828, by Volpi and Benvenuti (2003: 72).

†*Isaura* Agassiz, 1844: 14. Unjustified emendation and objective synonym of of *Isaurus* Gray, 1828, and junior homonym of *Isaura* Lamouroux, in Audouin, Bourdon, de Candolle, d’Aubebard de Férussac, Deshayes, Deslongchamps, É. Geoffroy Saint-Hilaire, I. Geoffroy Saint-Hilaire, Guérin, Guillemin, de Jussieu, Kunth, Delafosse, Lamouroux, Latreille, Prévost, Richard & Bory de Saint-Vincent, 1826. See *Isaurus* Gray, 1828.

†*Isaura* Lamouroux, in Audouin, Bourdon, de Candolle, d’Aubebard de Férussac, Deshayes, Deslongchamps, É. Geoffroy Saint-Hilaire, I. Geoffroy Saint-Hilaire, Guérin, Guillemin, de Jussieu, Kunth, Delafosse, Lamouroux, Latreille, Prévost, Richard & Bory de Saint-Vincent, 1826: 23. Type species *Isaurus tuberculatus* Gray, 1828, by subsequent designation by Haddon and Shackleton (1891b: 682); gender masculine. An objective synonym of *Isaurus* Gray, 1828 (see Low and Reimer 2012b: 46). See *Isaurus* Gray, 1828.

****Isaurus*** Gray, 1828: 8. Type species *Isaurus tuberculatus* Gray, 1828, by subsequent designation by Haddon and Shackleton (1891b: 682); gender masculine. The name *Isaurus* Gray, 1828, is a replacement name for *Isaura* Lamouroux, in Audouin, Bourdon, de Candolle, d’Aubebard de Férussac, Deshayes, Deslongchamps, É. Geoffroy Saint-Hilaire, I. Geoffroy Saint-Hilaire, Guérin, Guillemin, de Jussieu, Kunth, Delafosse, Lamouroux, Latreille, Prévost, Richard and Bory de Saint-Vincent, 1826, which Gray (1828: 8) believed to be preoccupied (see Low and Reimer 2012b: 46). Low and Reimer (2012b: 47) reversed the precedence of *Isaura* Lamouroux, in Audouin, Bourdon, de Candolle, d’Aubebard de Férussac, Deshayes, Deslongchamps, É. Geoffroy Saint-Hilaire, I. Geoffroy Saint-Hilaire, Guérin, Guillemin, de Jussieu, Kunth, Delafosse, Lamouroux, Latreille, Prévost, Richard & Bory de Saint-Vincent, 1826, and *Isaurus* Gray, 1828, thereby maintaining current and prevailing usage of the latter name. See also the synonyms *Antinedia* Duchassaing de Fonbressin & Michelotti, 1864, *Pales* Gray, 1867, Palythoa (Monothoa) Andres, 1883, *Panceria* Andres, 1877, and Zoanthus (Monanthus) Andres, 1883.

****Isozoanthus*** Carlgren, in Chun, 1903: 520. Type species *Isozoanthus giganteus* Carlgren, in Chun, 1903, by monotypy; gender masculine. Williams (2000) enacted Article 23.9 of the Code (ICZN 1999: 27, 28) to reverse precedence of Palythoa (Taeniothoa) Andres, 1883, and *Isozoanthus* Carlgren, in Chun, 1903. See also Palythoa (Taeniothoa) Andres, 1883.

****Kauluzoanthus*** Sinniger, Ocaña & Baco, 2013: 8. Type species *Kauluzoanthus kerbyi* Sinniger, Ocaña & Baco, 2013, by original designation; gender masculine.

****Kulamanamana*** Sinniger, Ocaña & Baco, 2013: 4. Type species *Kulamanamana haumaeaae* Sinniger, Ocaña & Baco, 2013, by original designation; gender feminine.

†*Lirrevia* Delphy, 1939: 270. Replacement name for *Verrillia* Andres, 1883; gender feminine. *Lirrevia* Delphy, 1939, and *Verrillia* Andres, 1883, are thus subjective junior synonyms of *Epizoanthus* Gray, 1867. See also *Verrillia* Andres, 1883.

†*Mammillifera* [*sic*]. Incorrect spelling of *Mammillifera* Le Sueur, 1817, by Blainville (1830: 295).

†*Mammillifera* [*sic*]. Incorrect spelling of *Mammillifera* Le Sueur, 1817, by Gistel (1848: 181).

†*Mammillifera* Le Sueur, 1817: 177. Type species *Mammillifera auricula* Le Sueur, 1817, by subsequent designation by Haddon and Shackleton (1891a: 626); gender feminine. The type species is now assigned to the genus *Zoanthus* Lamarck, 1801 (see Appendix 3), making *Mammillifera* Le Sueur, 1817, a junior subjective synonym of *Zoanthus* Lamarck, 1801. See also *Actimastus* Rafinesque, 1818.

†*Mammithoa* Andres, 1883a: 521. Type species *Mammillifera nymphaea* Le Sueur, 1817, herein designated (see Appendix 3); gender feminine. First proposed as a subgenus of *Palythoa* Lamouroux, 1816. The type species is now a junior subjective synonym of *Zoanthus pulchellus* Duchassaing de Fonbressin & Michelotti, 1864, which is currently assigned to the genus *Zoanthus* Lamarck, 1801 (see Appendix 3), making Palythoa (Mammithoa) Andres, 1883, a junior subjective synonym of *Zoanthus* Lamarck, 1801. See also the incorrect original spelling *Mammothoa* Andres, 1883.

†*Mammithoa* [*sic*]. An incorrect original spelling of *Mammithoa* Andres, 1883, by Andres (1883a: 533–535; 1883b: 325–327; 1884: 318–320) (see Appendix 3).

†*Mardoell* Danielssen, 1890: 117–126. Type species *Mardoell erdmanni* Danielssen, 1890, by monotypy; gender feminine. The type species is assigned to the genus *Epizoanthus* Gray, 1867 (see Low and Reimer 2012a: 85, Lwowsky 1913: 603, 604), making *Mardoell* Danielssen, 1890, a junior subjective synonym of *Epizoanthus* Gray, 1868. See also the incorrect emendation *Mardoellia* Blanchard, 1893.

†*Mardoell* [*sic*]. Incorrect spelling of *Mardoell* Danielssen, 1890, by Bell (1906: 762) and also in Neave’s (1940: 43) *Nomenclator zoologicus* entry.

†*Mardoellia* Blanchard, 1893: 130. Type species *Mardoell erdmanni* Danielssen, 1890, by monotypy; gender feminine. Blanchard (1893: 130) incorrectly emended the genus-group name *Mardoell* Danielssen, 1890, to *Mardoellia* (see Low and Reimer 2012a: 84). *Mardoellia* Blanchard, 1893, is therefore a junior objective synonym of *Mardoell* Danielssen, 1890, and a junior subjective synonym of *Epizoanthus* Gray, 1867. See also *Mardoell* Danielssen, 1890.

****Mesozoanthus*** Sinniger & Häussermann, 2009: 31, 32. Type species *Mesozoanthus fossii* Sinniger & Häussermann, 2009, by original designation and monotypy; gender masculine.

*Meto* Gistel, 1848: 181. Replacement name for *Hughea* Lamouroux, 1821. Not a zoantharian (see remarks under *Hughea* Lamouroux, 1821).

****Microzoanthus*** Fujii & Reimer, 2011: 421. Type species *Microzoanthus occultus* Fujii & Reimer, 2011, by original designation; gender masculine.

†*Monanthus* Andres, 1883a: 538, 540, 541, 543. Type species *Isaurus tuberculatus* Gray, 1828, herein designated (see Appendix 3); gender masculine. First proposed as a subgenus of *Zoanthus* Lamarck, 1801. The type species is also the type species of *Isaurus* Gray, 1828, making Zoanthus (Monanthus) Andres, 1883, a junior objective synonym of *Isaurus* Gray, 1828 (see Appendix 3).

†*Monothoa* Andres, 1883a: 521. Type species *Panceria spongiosa* Andres, 1877, herein designated (see Appendix 3); gender feminine. The type species is now a junior subjective synonym of *Isaurus tuberculatus* Gray, 1828, (see Appendix 3), making Palythoa (Monothoa) Andres, 1883, a junior subjective synonym of *Isaurus* Gray, 1828.

*Montlivaltia* [*sic*]. Incorrect subsequent spelling of *Montlivaltia* Lamouroux, 1821, by Ehrenberg (1834a: 271).

*Montlivaltia* Lamouroux, 1821: 78. Type species *Montlivaltia caryophyllata* Lamouroux, 1821, by monotypy; gender feminine. Lamouroux (1821: 78) and Audouin (1826: 229) discussed the similarities of *Montlivaltia* Lamouroux, 1821, and *Palythoa* Lamouroux, 1816. *Montlivaltia* Lamouroux, 1821, is now considered to be a species of Scleractinia (see Stolarski and Roniewicz 2001: 1097).

****Nanozoanthus*** Fujii & Reimer, 2013: 512. Type species *Nanozoanthus harenaceus* Fujii & Reimer, 2013, by original designation; gender masculine.

****Neozoanthus*** Herberts, 1972: 137. Type species *Neozoanthus tulearensis* Herberts, 1972, by original designation and monotypy; gender masculine.

*Orinia* Duchassaing de Fonbressin & Michelotti, 1860: 54. Type species *Orinia torpida* Duchassaing de Fonbressin & Michelotti, 1860, by monotypy; gender feminine. The type species is considered to be a junior subjective synonym of *Actinia osculifera* Le Sueur, 1817 (see Cha 2007: 40; Fautin 2016: 38). As the type species of *Orinia* Duchassaing de Fonbressin & Michelotti, 1860, is currently a junior subjective synonym of a species assigned to *Rhodactis* Milne Edwards & Haime, 1851 (see Fautin 2016: 38), the former becomes a junior subjective synonym of the latter.

****Palaeozoanthus*** Carlgren, 1924: 470–473. Type species *Palaeozoanthus reticulatus* Carlgren, 1924, by original designation and monotypy; gender masculine.

†*Pales* Gray, 1867: 234, 235. Type species *Pales cliftoni* Gray, 1867, by monotypy; gender masculine. A subjective junior synonym of *Isaurus* Gray, 1828 (see Muirhead and Ryland 1985: 325). The genus-group name *Pales* Gray, 1867, is also a junior homonym of *Pales* Meigen, 1800 (Diptera), *Pales* Robineau-Desvoidy, 1830 (Diptera), and *Pales* Koch, 1850 (Arachnida).

****Palythoa*** Lamouroux, 1816: 359. Type species *Palythoa* [*sic*] *stellata* Lamouroux, 1816 [= *Alcyonium mammillosum* Ellis, in Ellis and Solander, 1786], subsequent designation by Haddon and Shackleton (1891b: 691); gender feminine. See Appendix 3 for a discussion on the type species and its designation. Frequently incorrectly spelt as “Palythoa” (see Low and Reimer 2011b: 63). See also the synonyms *Cavolinia* Schweigger, 1819, *Corticifera* Le Sueur, 1817, *Cynicus* Gistel, 1848, Palythoa (Corticithoa) Andres, 1883, Palythoa (Gemmithoa) Andres, 1883, and the incorrect spellings *Palythoa* and *Polythoa*.

†Palythoa (Corticithoa) Andres, 1883. See *Corticithoa* Andres, 1883.

†Palythoa (Endeithoa) Andres, 1883. See *Endeithoa* Andres, 1883.

†Palythoa (Gemmithoa) Andres, 1883. See *Gemmithoa* Andres, 1883.

†Palythoa (Mammithoa) Andres, 1883. See *Mammithoa* Andres, 1883.

†Palythoa (Mammothoa) Andres, 1883. See *Mammothoa* Andres, 1883, and *Mammithoa* Andres, 1883.

†Palythoa (Monothoa) Andres, 1883. See *Monothoa* Andres, 1883.

†Palythoa (Taeniothoa) Andres, 1883. See *Taeniothoa* Andres, 1883.

†*Palythoaster* Haeckel, 1875: 44, pl. 1, fig. 5. Type species *Palythoa savignyi* Audouin, 1826, by monotypy; gender masculine. The type species *Palythoa savignyi* Audouin, 1826, is currently assigned to the genus *Palythoa* Lamouroux, 1816, making *Palythoaster* Haeckel, 1875, a junior subjective synonym of *Palythoa* Lamouroux, 1816 (see Appendix 3).

†*Palythoa* [*sic*]. An incorrect spelling of *Palythoa* Lamouroux, 1816 (see Low and Reimer 2011b: 63). See also *Palythoa* Lamouroux, 1812.

*Palythoa* Lamouroux, 1812: 188. Type species *Gorgonia muricata* Pallas, 1766, by subsequent designation by Low and Reimer (2011b: 64); gender not determined. Now a subjective synonym of *Muricea* Lamouroux, 1821 [Octocorallia] (Low and Reimer 2011b: 64). Not a zoantharian (Low and Reimer 2011b: 63). *Palythoa* is also sometimes used as an incorrect spelling of *Palythoa* Lamouroux, 1816 (see Low and Reimer 2011b: 63).

†*Palythoa* [*sic*]. Incorrect spelling of *Palythoa* Lamouroux, 1816, by various authors (e.g., Milliman et al. 1974: 162).

†*Panceria* Andres, 1877: 221–226. Type species *Panceria spongiosa* Andres, 1877, by monotypy; gender feminine. The type species is a subjective synonym of *Isaurus tuberculatus* Gray, 1828 (see Appendix 3), making *Panceria* Andres, 1877, a junior subjective synonym of *Isaurus* Gray, 1828.

†*Parapalythoa* Verrill, 1900: 560. Type species *Parapalythoa heilprini* Verrill, 1900, by monotypy. Herein considered to be a junior subjective synonym of *Palythoa* Lamouroux, 1816 (see Appendix 3). See also *Protopalythoa* Verrill, 1900.

****Parazoanthus*** Haddon and Shackleton, 1891a: 653, 654. Type species *Palythoa axinellae* O. Schmidt, 1862, by original designation; gender masculine. See also the synonyms *Bergia* Duchassaing de Fonbressin & Michelotti, 1860, *Gemmaria* Duchassaing de Fonbressin & Michelotti, 1860, *Heterozoanthus* Verrill, 1870, *Parapalythoa* Verrill, 1900, and *Protopalythoa* Verrill, 1900.

*Peachia* Gosse, 1855: 270, 271. Type species *Peachia hastata* Gosse, 1855, by subsequent designation by Carlgren (1949: 32); gender feminine. Not a zoantharian (Fautin et al. 2007: 220, 221; Fautin 2016: 47, 117).

*Platyzoanthus* Saville-Kent, 1893: 155. Type species *Platyzoanthus mussoides* Saville-Kent, 1893, by monotypy; gender masculine. Not a zoantharian (see Haddon 1898: 409, den Hartog 1980: 37, 39; Fautin 2016, 25, 28).

†*Palythoa* [*sic*]. Incorrect spelling of *Palythoa* Lamouroux, 1816, by Danielssen (1890: 136). The Danish version of the same text on the same page is spelt “*Polythoa*” (see *Polythoa*).

†*Palythoa* [*sic*]. Incorrect spelling of *Palythoa* Lamouroux, 1816, by Gistel (1848: 181).

†*Palythoa* [*sic*]. Incorrect spelling of *Palythoa* Lamouroux, 1816, by Schweigger (1819: 100). Also throughout Andres (1883a, b, 1884).

†*Protopalythoa* Verrill, 1900: 562. Replacement name for *Gemmaria* Duchassaing de Fonbressin & Michelotti, 1860, preoccupied by *Gemmaria* McCrady, 1859 (Hydrozoa). Type species *Gemmaria rusei* Duchassaing de Fonbressin & Michelotti, 1860, by typification of the preoccupied name; gender feminine. Verrill’s (1900: 562) original designation of *Gemmaria variabilis* Duerden, 1898, as the type species of this genus-group name is invalid (see Appendix 3). See also *Gemmaria* Duchassaing de Fonbressin & Michelotti, 1860.

†*Rhyzanthus* Andres, 1883a: 538, 541–544. Type species *Actinia sociata* Ellis, 1768, herein designated (see Appendix 3); gender masculine. First proposed as a subgenus of *Zoanthus* Lamarck, 1801. The type species is also the type species of the genus *Zoanthus* Lamarck, 1801, making Zoanthus (Rhyzanthus) Andres, 1883, a junior objective synonym of *Zoanthus* Lamarck, 1801.

†*Savaglia* Nardo, 1877: 674. Unjustified emendation of *Savalia* Nardo, 1844 (see Appendix 3). See *Savalia* Nardo, 1844.

****Savalia*** Nardo, 1844: 433, 434. Type species *Gorgonia savaglia* Bertolini, 1819, by monotypy; gender feminine. *Savalia savaglia* (Bertolini, 1819), and *Gerardia lamarcki* (Haime, 1849), are currently considered subjective synonyms (see Appendix 3). As each species is also the type species of its respective genus, the genera *Savalia* Nardo, 1844, and *Gerardia* Lacaze-Duthiers, 1864, are also subjective synonyms. As discussed in Poche (1914), the valid name for this taxon is *Savalia savaglia* (Bertolini, 1819), which agrees with the Principle of Priority (Article 23, ICZN 1999: 24) (see discussion in Appendix 3). The name *Savaglia* is an unjustified emendation of *Savalia* Nardo, 1844, by Nardo (1877).

*Scolanthus* Gosse, 1853: 157. Type species *Scolanthus callimorphus* Gosse, 1853, by monotypy. Not a zoantharian (see Manuel 1981: 266; Fautin 2016: 46, 129).

†*Sidisia* Gray, 1858: 489. Type species *Sidisia barleei* Gray, 1859, by subsequent monotypy; gender feminine. *Sidisia* Gray, 1858, was first proposed without the inclusion of any nominal species. In a subsequent paper, Gray (1859: 532) included *Sidisia barleei* Gray, 1859, as the only species in *Sidisia* Gray, 1858, and *Sidisia barleei* Gray, 1859, becomes the type species of *Sidisia* Gray, 1858, by subsequent monotypy (Article 69.3, ICZN 1999: 73). Subjective synonym of *Epizoanthus* Gray, 1867. *Sidisia* Gray, 1858, was suppressed in favour of *Epizoanthus* Gray, 1867 (see Opinion 1689, ICZN 1992: 236).

*Scolanthus* [*sic*]. Incorrect spelling of *Scolanthus* Gosse, 1853, by Gray (1867: 240).

†*Sphenopus* [*sic*]. Incorrect spelling of *Sphenopus* Steenstrup, 1856, by Herberts (1972: 72, 80, 142).

†*Sphenopus* [*sic*]. Incorrect spelling of *Sphenopus* Steenstrup, 1856, by Long, Poiner and Wassenberg (1995: 134).

****Sphenopus*** Steenstrup, 1856: 37. Type species *Sabella marsupialis* Gmelin, 1791, by original designation; gender masculine.

†*Stephanidium* Hertwig, 1888: 52. Type species *Stephanidium schulzii* Hertwig, 1888, by monotypy; gender neuter. From the original description, the type species is possibly a species of zoantharian, but the type material will need to be located and examined (also see Delage and Hérouard 1901: 662). Herein, we make no decision as to the validity of this genus and species in the event that the identification of this genus and species, but note that this genus-group name is preoccupied by *Stephanidium* Ehrenberg, 1839 (*incerta sedis*).

†*Taeniothoa* Andres, 1883a: 521, 532. Type species *Zoanthus sulcatus* Gosse, 1859, by subsequent designation by Williams (2000: 193); gender feminine. The type species is now assigned to the genus *Isozoanthus* Carlgren, in Chun, 1903 (see Williams 2000: 195), making Palythoa (Taeniothoa) Andres, 1883, a subjective synonym of *Isozoanthus* Carlgren, in Chun, 1903. Williams (2000: 195) enacted Article 23.9 (ICZN 1999: 27, 28) to reverse precedence between these genus-group names, thereby making Palythoa (Taeniothoa) Andres, 1883, a *nomen oblitum*, and *Isozoanthus* Carlgren, in Chun, 1903, a *nomen protectum*.

****Terrazoanthus*** Reimer & Fujii, 2010: 20. Type species *Terrazoanthus onoi* Reimer & Fujii, 2010, by original designation; gender masculine.

****Thoracactis*** Gravier, 1918: 12. Type species *Thoracactis topsenti* Gravier, 1918, by monotypy; gender masculine. Gravier (1918: 12, footnote) stated that the etymology of this name was from the Greek word “θοραξ, αχος” (= breastplate). It is also clear that Gravier (1918: 12) was using this word in combination with the ending “-*actis*” as commonly used for many other genera of Anthozoa, sea anemones in particular (e.g., *Amphiactis*, *Calliactis*, *Epiactis*, *Monactis*, *Paractis*; see Fautin 2016 for additional examples). In this context, the genus-group names ending with this suffix are to be treated in the same way as *Zoantha* and *Zoanthus* (see Low and Reimer 2012a: 85) in which Article 30.1.3 of the Code (ICZN 1999: 35) applies. The emendation of this name to *Thoracactus* by Walsh (1967: 49) is thus not justified. Also see the unjustified emendation *Thoracactis* by Walsh (1967: 49).

†*Thoracactus* Walsh, 1967: 49. Unjustified emendation (and junior objective synonym) of *Thoracactis* Gravier, 1918.

†*Thoracactis* [*sic*]. Incorrect spelling of *Thoracactis* Gravier, 1918, by Herberts (1972: 80).

*Triga* Gray, 1867: 239. Type species *Triga philippinensis* Gray, 1867, by monotypy; gender feminine. McMurrich (1889: 125) stated that “in all probability this is a *Gemmaria*”, considering it to be similar to *Gemmaria rusei* Duchassaing de Fonbressin and Michelotti, 1860, which is clearly a species of *Palythoa* Lamouroux, 1812. Heider (1899a: 283; 1899b: 133) cited McMurrich (1889) and agreed, placing this species in *Gemmaria*. Ryland and Lancaster (2003: 411) discussed that “[t]he solitary *Triga philippinesis* Gray, 1867, though tentatively referred to *Gemmaria* by McMurrich (1889), seems more likely to have been an actinian, but Gray’s [1867] two-line diagnosis is insufficient even to determine the order with any certainty”. The solitary and long polyps of this species would indicate a species belonging to the genus *Sphenopus* Steenstrup, 1856. The final identity of this genus-group name and its type species remain unresolved.

****Umimayanthus*** Montenegro, Sinniger & Reimer, 2015: 76. Type species *Umimayanthus chanpuru* Montenegro, Sinniger & Reimer, 2015, by original designation; gender masculine.

†*Verrillia* Andres, 1883a: 520, 545. Type species *Epizoanthus crassus* Verrill, 1869, by monotypy; gender feminine. The type species is currently assigned to the genus *Epizoanthus* Gray, 1867, making *Verrillia* Andres, 1883, a junior subjective synonym of *Epizoanthus* Gray, 1867 (see Appendix 3). *Verrillia* Andres, 1883, is preoccupied by *Verrillia* Stearns, 1873 (Scleractinia), and the name *Lirrevia* was proposed by Delphy (1939: 270) was proposed as a replacement name. See also *Lirrevia* Delphy, 1939.

****Zibrowius*** Sinniger, Ocaña & Baco, 2013: 7. Type species *Zibrowius ammophilus* Sinniger, Ocaña & Baco, 2013, by original designation; gender masculine.

†*Zoantha* Lamarck, 1801: 363. Type species *Actinia sociata* Ellis, 1768, by monotypy; gender feminine. This is an incorrectly Latinised spelling of a name derived from Greek and should be corrected to *Zoanthus* (see Article 30.1.3, ICZN 1999: 35). This name was first correctly emended by Tilesius (1809: 374, footnote) (see Low and Reimer 2012a: 85). See also *Zoanthus* Lamarck, 1801.

†*Zoanthella* van Beneden, 1897: 196. No type species designated. This name was established for zoantharian larvae of and is no longer in use as genus-group name (see Ryland et al. 2000: 191). Nevertheless, this name remains nomenclaturally available despite still being used as name for larvae.

†*Zoanthina* van Beneden, 1897: 200. No type species designated. This name was established for zoantharian larvae of and is no longer in use as genus-group name (see Ryland et al. 2000: 191). Nevertheless, this name remains nomenclaturally available despite still being used as name for larvae.

****Zoanthus*** Lamarck, 1801: 363. Type species *Actinia sociata* Ellis, 1768, by monotypy; gender masculine. The name *Zoanthus* is derived from the Greek words ζωο (zoo = animal) and ανθος (anthos = flower). According to Article 30.1.3 (ICZN 1999: 35), the Greek ending “-os” should be latinised to the Latin masculine -*us.* Cuvier (1800: tables 9, 10) first used the name “*Zoanthus*”, but without the inclusion of species or a description and is a *nomen nudum*. Lamarck (1801: 363) next used the name *Zoantha* with the inclusion of *Actinia sociata* Ellis, 1768, the type species by monotypy. It is possible that Lamarck (1801) assumed that the gender of the Greek word for flower was feminine. Tilesius (1809: 394, footnote) first emended Lamarck’s (1801) name to *Zoanthus*. This emendation is conventionally attributed to Cuvier (1816: 53) (see also Haddon and Shackleton 1891b, 676; Low and Reimer 2012a: 85). See also the synonyms *Actimastus* Rafinesque, 1818, *Actinorhyza* Blainville, 1830, *Anthozoon* Gistel, 1848, *Mammillifera* Le Sueuer, 1817, Palythoa (Mammithoa) Andres, 1883, Zoanthus (Rhyzanthus) Andres, 1883, and incorrect original spelling *Zoantha* Lamarck, 1801.

†Zoanthus (Corticanthus) Andres, 1883. See *Corticanthus* Andres, 1883.

†Zoanthus (Monanthus) Andres, 1883. See *Monanthus* Andres, 1883.

†Zoanthus (Rhyzanthus) Andres, 1883. See *Rhyzanthus* Andres, 1883.

†*Zoanthus* [*sic*]. Incorrect spelling of *Zoanthus* Lamarck, 1801 (e.g. Carlos et al. 1999: 1057, Kenny 2008: 78, Untawale and Dhargalkar 2002: 113).

## Appendix 3.

Taxonomic and nomenclatural remarks on some previous unidentified or problematic family- and genus-group names in the Zoantharia Rafinesque, 1815

In this section, previously unidentified (or problematic) genus-group names have been grouped according to the senior synonym that they have been identified with. Two problematic groups of genus-group names are discussed last: 1) *Gemmaria* Duchassaing de Fonbressin & Michelotti, 1860, *Parapalythoa* Verrill, 1900, *Protopalythoa* Verrill, 1900, and *Haplotella* Stechow, 1919; and 2) *Savalia* Nardo, 1844, *Savaglia* Nardo, 1877, and *Gerardia* Lacaze-Duthiers, 1864.

### Synonyms of *Epizoanthus* Gray, 1867 (Epizoanthidae) (I): *Corticanthus* Andres, 1883, and *Endeithoa* Andres, 1883

The genus-group name *Corticanthus* was proposed as a subgenus of *Zoanthus* Lamarck, 1801 by Andres (1883a: 538, 541, 544; 1883b: 330, 333, 336; 1884: 323, 326, 327, 329) with the inclusion of *Epizoanthus paguriphilus* Verrill, 1882, *Mammillifera anduzii* Duchassaing de Fonbressin & Michelotti, 1860, *Mammillifera conferta* Verrill, 1869, and *Mammillifera nitida* Verrill, 1869. *Epizoanthus paguriphilus*, is herein designated as the type species of Zoanthus (Corticanthus) Andres, 1883, making this genus-group name a subjective junior synonym of *Epizoanthus* Gray, 1867, as the type species is now assigned to the genus *Epizoanthus* (Pax and Müller 1956: 12, 13; Reimer et al. 2010b: 730, 733, Walsh 1967: 45, 46).

The genus-group name *Endeithoa* was proposed as a subgenus of *Palythoa* by Andres (1883a: 521, 531; 1883b: 313, 323; 1884: 307, 316, 317) with the inclusion of *Zoanthus norvegicus* Koren & Danielssen, 1877, and *Zoanthus rubricornis* Holdsworth, 1861. *Zoanthus norvegicus* is herein designated as the type species of Polythoa (Endeithoa) making this genus-group name a subjective junior synonym of *Epizoanthus*, as the type species is now assigned to the genus *Epizoanthus* Gray, 1867 (Carreiro-Silva et al. 2011: 408, 413, Walsh 1967: 45).

### Synonyms of *Epizoanthus* Gray, 1867 (Epizoanthidae) (II): *Lirrevia* Delphy, 1939, and *Verrillia* Andres, 1883

The genus-group name *Verrillia* was proposed by Andres (1883a: 520, 545; 1883b: 312, 337; 1884: 306, 330, 331) with the inclusion of only *Epizoanthus crassus* Verrill, 1869, the type species by monotypy (Article 68.3 of the Code; ICZN 1999: 71). *Epizoanthus crassus* Verrill, 1869, is currently assigned to the genus *Epizoanthus* Gray, 1867 (Lwowsky 1913: 560, Walsh 1967: 39), and *Verrillia* is thus a junior subjective synonym of *Epizoanthus* Gray, 1867. *Verrillia* Andres, 1883, is not a valid name as it is a junior homonym of *Verrillia* Stearns, 1873, which was proposed for a genus of scleractinian coral. Delphy (1939: 269, 270) proposed the replacement name *Lirrevia*, which is an objective synonym of *Verrillia* Andres, 1883, and a junior subjective synonym of *Epizoanthus* Gray, 1867.

### Synonyms of *Isaurus* Gray, 1828 (Zoanthidae): *Monanthus* Andres, 1883, *Monothoa* Andres, 1883, and *Panceria* Andres, 1877

Muirhead and Ryland (1985: 325) listed Polythoa (Monothoa) Andres, 1883, Zoanthus (Monanthus) Andres, 1883, and *Panceria* Andres, 1877, as synonyms of *Isaurus* Gray, 1828, but did not give details for this synonymy. Herein, type species are designated for Polythoa (Monothoa) Andres, 1883, and Zoanthus (Monanthus) Andres, 1883, to formally fix the identity of these genus-group names. The nomenclatural consequences of our type species designations are in agreement with the taxonomic conclusions of Muirhead and Ryland (1985).

The genus-group name *Monothoa* was proposed as a subgenus of *Palythoa* Lamouroux, 1816, by Andres (1883a: 521, 530, 537; 1883b: 313, 322, 329; 1884: 307, 315, 316, 322) with the inclusion of *Hughaea caraibeorum* Duchassaing de Fonbressin, 1850, *Mamillifera fulva* Quoy & Gaimard, 1834, *Mamillifera vanikorensis* Quoy & Gaimard, 1834, *Mamillifera viridifusca* Quoy & Gaimard, 1834, *Panceria spongiosa* Andres, 1877, and *Triga philippinensis* Gray, 1867. *Panceria spongiosa* Andres, 1877, is herein designated as the type species of Polythoa (Monothoa) Andres, 1883. *Panceria spongiosa* Andres, 1877, is currently considered to be a junior subjective synonym of *Isaurus tuberculatus* Gray, 1828 (Muirhead and Ryland 1985: 326), and Polythoa (Monothoa) Andres, 1883, becomes a junior subjective synonym of *Isaurus* Gray, 1828.

The genus-group name *Monanthus* was proposed as a subgenus of *Zoanthus* Lamarck, 1801, by Andres (1883a: 538, 540, 541, 543; 1883b: 330, 332, 333, 335; 1884: 323, 325, 326, 328, 329) with the inclusion of *Isaurus tuberculatus* Gray, 1828, *Isaura neglecta* Duchassaing de Fonbressin and Michelotti, 1860, *Pales cliftoni* Gray, 1867, and *Palythoa savignyi* Audouin, 1826. *Isaurus tuberculatus* Gray, 1828, is herein designated as the type species of Zoanthus (Monanthus) Andres, 1883, thereby making Zoanthus (Monanthus) Andres, 1883, a junior objective synonym of *Isaurus* Gray, 1828, as they have the same type species (Article 61.3.3 of the Code, ICZN 1999: 64) (also see Low and Reimer 2012b: 47).

The genus-group name *Panceria* was proposed by Andres (1877: 221, 226) with the inclusion of *Panceria spongiosa* Andres, 1877, the type species by monotypy (Article 68.3 of the Code, ICZN 1999: 71). As discussed above, *Panceria spongiosa* Andres, 1877, is a junior subjective synonym of *Isaurus tuberculatus* Gray, 1828 and *Panceria* Andres, 1877, becomes a junior subjective synonym of *Isaurus* Gray, 1828.

### Synonyms of *Palythoa* Lamouroux, 1816 (Sphenopidae) (I): *Cavolinia* Schweigger, 1819, and *Cynicus* Gistel, 1848

Schweigger (1819: 99, 100) proposed the genus-group name *Cavolinia* for two species of zoanthids: *Alcyonium mammillosum* Ellis, in Ellis & Solander, 1786 (incorrectly spelt as “*mamillosum*”), and *Cavolinia rosea* Schweigger, 1819 (an unnecessary replacement name for *Madrepora denudata* Cavolini, 1785). *Alcyonium mammillosum* Ellis, in Ellis & Solander, 1786, and *Madrepora denudata* Cavolini, 1785 (and therefore *Cavolinia rosea* Schweigger, 1819), are now assigned to the genus *Palythoa* Lamouroux, 1816 (see Milne Edwards 1857: 301, Ryland and Lancaster 2003: 410), making *Cavolinia* Schweigger, 1819, a junior subjective synonym of *Palythoa* Lamouroux, 1816.

No type species has been designated for *Cavolinia* Schweigger, 1819, and no designation is necessary as *Cavolinia* Schweigger, 1819, is an invalid junior homonym of *Cavolinia* Abildgaard, 1791 (Mollusca), and *Cavolinia* Bruguière, 1791 (Mollusca), and has been placed on the *Official Index of Rejected and Invalid Generic Names in Zoology* (see ICZN 1969: 28).

The replacement name *Cynicus* was proposed by Gistel (1848: viii) for *Cavolinia* Schweigger, 1819. *Cynicus* Gistel, 1848, is therefore an unnecessary replacement name for, and junior objective synonym of, *Cavolinia* Schweigger, 1819.

### Synonyms of *Palythoa* Lamouroux, 1816 (Sphenopidae) (II): *Corticifera* Le Sueur, 1817, *Corticithoa* Andres, 1883, and *Gemmithoa* Andres, 1883

The genus-group name *Corticifera* was proposed by Le Sueur (1817: 178, 179) with the inclusion of *Corticifera flava* Le Sueur, 1817, and *Corticifera glareola* Le Sueur, 1817. Haddon and Shackleton (1891b: 692) designated *Corticifera glareola* Le Sueur, 1817, as the type species of *Corticifera* Le Sueur, 1817. As the type species of *Corticifera*, is currently assigned to genus *Palythoa*, *Corticifera*, is now a junior subjective synonym of *Palythoa* (see also Haddon and Shackleton 1891b: 692).

The genus-group name *Corticithoa* was proposed as a subgenus of *Palythoa* by Andres (1883a: 521, 535–538; 1883b: 313, 327–330; 1884: 307, 320–323) with the inclusion of *Alyconium tuberculosum* Esper, 1805, *Corticifera aggregata* Lesson, 1830, *Corticifera glareola* Le Sueur, 1817, *Gemmaria humilis* Verrill, 1869, *Mammillifera clavata* Duchassaing de Fombressin, 1850, *Mamillifera lutea* Quoy & Gaimard, 1834, and *Palythoa cinerea* Duchassaing de Fonbressin & Michelotti, 1864. *Alyconium tuberculosum* Esper, 1805, is herein designated as the type species of Palythoa (Corticithoa) Andres, 1883. *Alyconium tuberculosum*, is currently assigned to the genus *Palythoa* (see Reimer et al. 2006: 92, Ryland and Lancaster 2003: 409, 410, Walsh 1967: 19, 20). By this type species designation, Palythoa (Corticithoa) Andres, 1883, becomes a junior subjective synonym of *Palythoa*.

The genus-group name *Gemmithoa* was proposed as a subgenus of *Palythoa*, by Andres (1883a: 521, 532, 533; 1883b: 313, 324, 325; 1884: 307, 318) with the inclusion of *Mammillifera brevis* Duchassaing de Fonbressin, 1850, the type species by monotypy (Article 68.3 of the Code; ICZN 1999: 71). *Mammillifera brevis* Duchassaing de Fombressin, 1850, is now assigned to the genus *Palythoa* (see Walsh 1967: 5), making Polythoa (Gemmithoa) Andres, 1883, a junior subjective synonym of *Palythoa* Lamouroux, 1816.

### Synonyms of *Palythoa* Lamouroux, 1816 (Sphenopidae) (III): *Palythoaster* Haeckel, 1875

Haeckel (1875: 44, pl. 1, fig. 5) proposed the name *Palythoaster* for a single species, *Palythoa savignyi* Audouin, 1826, the type species by monotypy (Article 68.3 of the Code, ICZN 1999: 71). Although the date on the title-page of the work by Haeckel is “1876”, it was available in 1875 (see Leuckart 1875: 463).

Ehrenberg (1834a: 269; 1834b: 45) considered *Palythoa savignyi* Audouin, 1826, to be a valid species of zoantharian, but placed it in the genus *Hughea* Lamouroux, 1821. The genus *Hughea* Lamouroux, 1821, has long been confused for a genus of a zoantharian, which it is not (see Ryland and Lancaster 2003: 411; Appendix 2).

As discussed by Low and Reimer (2012b), the name *Palythoa savignyi* was proposed by Audouin (1826: 229) for an unnamed figured by Savigny (1811: pl. 2, fig. 1) from Egypt. Based on the figure and the other information provided by Savigny (1811) and Audouin (1826), we herein agree with the opinion of Ehrenberg (1834a: 269; 1834b: 45) in considering *Palythoa savignyi* Audouin, 1826, to be a valid species of zoantharian, but unlike this previous author, we consider it to be a species of *Palythoa* Lamouroux, 1816.

As the type species of the genus *Palythoaster* Haeckel, 1875, is considered to be a species of *Palythoa* Lamouroux, 1816, the former becomes a junior subjective synonym of the latter.

### Synonyms of *Zoanthus* Lamarck, 1801 (Zoanthidae) (I): *Actimastus* Rafinesque, 1818, and *Mammillifera* Le Sueur, 1817

The genus-group name *Mammillifera* was proposed by Le Sueur (1817: 177, 178) with the inclusion of *Mammillifera auricula* Le Sueur, 1817, and *Mammillifera nymphaea* Le Sueur, 1817. *Mammillifera auricula* was designated as the type species of *Mammillifera* (see Haddon and Shackleton 1891a: 626). As the type species *Mammillifera auricula* is currently assigned to the genus *Zoanthus* Lamarck, 1801 (Duerden 1898: 334, Walsh 1967: 22, Reimer et al. 2012a: 7), *Mammillifera* Le Sueur, 1817, and *Zoanthus* Lamarck, 1801, are subjective synonyms.

Rafinesque (1818: 271) proposed the name *Actimastus* to replace *Mammillifera* Le Sueur, 1817, stating that “*Mammillifera* of Lesueur, is rather too long; it is too much like *Mammillaria* in meaning and sense, and is composed of two Latin names united, which are tolerated in the specific nomenclature, but not often in the generic; lastly it has too much likeness to the classical name of Mammalia to be tolerated. It must then be changed into *Actimastus*; meaning radiated mammilla”. As it is an unjustified emendation of *Mammillifera* Le Sueur, 1817 (Article 19.1 of the Code, ICZN 1999: 21), *Actimastus* Rafinesque, 1818, becomes a junior objective synonym of *Mammillifera* Le Sueur, 1817.

### Synonyms of *Zoanthus* Lamarck, 1801 (Zoanthidae) (II): *Actinorhiza* Agassiz, 1846, and *Actinorhyza* Blainville, 1830

In a discussion on the genus *Zoanthus* Lamarck, 1801, Blainville (1830: 295) proposed the replacement name *Actinorhyza* for this genus-group stating only “[c]e genre, dans notre Système de nomenclature, pourroit ètre nommé Actinorhyse, *Actinorhyza*”. We herein consider the name *Actinorhyza* Blainville, 1830, to be an unnecessary replacement name for, and junior objective synonym of, *Zoanthus* Lamarck, 1801. The type species is thus identical to that of *Zoanthus* Lamarck, 1801 (viz., *Actinia sociata* Ellis, 1768) (Article 67.8 of the Code, ICZN 1999: 68). In a later publication, Blainville (1834: 329) spelt the name as *Actinorhysa*, an incorrect subsequent spelling. Another incorrect subsequent spelling is *Actinorrhyza*, as used by Ehrenberg (1834a: 269).

Agassiz (1846: 7) proposed the unjustified emendation *Actinorhiza* for *Actinorhyza* Blainville, 1830, and the former is a junior objective synonym of the latter (Article 19.1 of the Code, ICZN 1999: 21).

### Synonyms of *Zoanthus* Lamarck, 1801 (Zoanthidae) (III): *Mammithoa* Andres, 1883, “*Mammothoa*” Andres, 1883, and *Rhyzanthus* Andres, 1883

The genus-group name *Mammithoa* was proposed as a subgenus of *Palythoa* Lamouroux, 1816, by Andres (1883a: 521, 533–535; 1883b: 313, 325–327; 1884: 307, 318–320). In the preliminary introduction to the newly proposed genus-group names, Andres (1883a: 521; 1883b: 313; 1884: 307) listed the name as Polythoa (Mammithoa) and included six species under this grouping: *Mammillifera auricula* Lesueur, 1817, *Mamillifera cingulata* Quoy and Gaimard, 1834, *Mammillifera nymphaea* Le Sueur, 1817, *Mammillifera univittata* Lorenz, 1860, *Mamillifera viridis* Quoy and Gaimard, 1834.

In a more detailed listing of the species included in the genus-group, the name was spelt as “*Mammothoa*” (Andres 1883a: 533–535; 1883b: 325–327; 1884: 318–320). As the genus-group names *Mammithoa* and *Mammothoa* were both used in the same publication (Andres 1883a: 521, 533–535), they were made available simultaneously and are both available names. First reviser action is herein taken to select *Mammithoa* Andres, 1883, as the correct original spelling (Article 24.2.3 of the Code, ICZN 1999: 30).

In this detailed listing of the genus-group name *Mammithoa* (incorrectly spelt as *Mammothoa*), Andres (1883a: 534, 535) discussed the included species as discussed above but with the name “Polythoa (Mammithoa) nymphosa Dana”, being used in place of *Mammillifera nymphaea* Le Sueur, 1817. From the synonymy of “Polythoa (Mammithoa) nymphosa Dana”, it is clear that the species-group name “*nymphosa* Dana” was a replacement for *nymphaea* Le Sueur, 1817. Although the species-group name *nymphosa* was attributed to Dana (see also Walsh 1967: 28), this species-group name was never used by Dana, and the authorship of Polythoa (Mammithoa) nymphosa should be attributed to Andres (1883a: 534, 535), and the name is herein considered an unnecessary replacement name for (and objective synonym of) *Mammillifera nymphaea* Le Sueur, 1817.

*Mammillifera nymphaea* Le Sueur, 1817, is herein designated as the type species of Polythoa (Mammithoa) Andres, 1883. *Mammillifera nymphaea* Le Sueur, 1817, is currently considered to be a subjective synonym of *Zoanthus pulchellus* Duchassaing de Fonbressin & Michelotti, 1864 (Acosta et al. 2005: 160, Duerden 1898: 334, Reimer et al. 2012a: 7). *Zoanthus pulchellus* Duchassaing de Fonbressin & Michelotti, 1864, is currently assigned to the genus *Zoanthus* Lamarck, 1801 (Acosta et al. 2005: 160, Reimer et al. 2012a: 7, Walsh 1967: 29) and Polythoa (Mammithoa) Andres, 1883, therefore becomes a junior subjective synonym of *Zoanthus* Lamarck, 1801.

The genus-group name *Rhyzanthus* was proposed as a subgenus of *Zoanthus* Lamarck, 1801, by Andres (1883a: 538, 541–544; 1883b: 330, 334–336; 1884: 306, 330, 331) with the inclusion of *Actinia sociata* Ellis, 1768, *Zoanthus alderi* Gosse, 1859, *Zoanthus dubia* Lesueur, 1817, *Zoanthus mertensii* Brandt, 1835, and *Zoanthus solandri* Lesueur, 1817. *Actinia sociata* Ellis, 1768, is herein designated as the type species of Zoanthus (Rhyzanthus) Andres, 1883, thereby making this genus-group name an objective junior synonym of *Zoanthus* Lamarck, 1801, as they have the same type species (Article 61.3.3 of the Code, ICZN 1999: 64).

### *Gemmaria* Duchassaing de Fonbressin and Michelotti, 1860, and its replacement names *Protopalythoa* Verrill, 1900, and *Haplotella* Stechow, 1919, junior subjective synonyms of *Palythoa* Lamouroux, 1816, as well as comments on *Parapalythoa* Verrill, 1900

The genus-group name *Gemmaria* was first proposed by Duchassaing de Fonbressin and Michelotti (1860: 55) with the inclusion of four nominal species: *Gemmaria rusei* Duchassaing de Fonbressin and Michelotti, 1860, *Gemmaria swiftii* Duchassaing de Fonbressin and Michelotti, 1860, *Mammillifera brevis* Duchassaing de Fonbressin, 1850, and *Mammillifera clavata* Duchassaing de Fonbressin, 1850. No type species was designated.

The identity of *Gemmaria* Duchassaing de Fonbressin & Michelotti, 1860, was debated by early workers. Duerden (1898: 353; 1903: 501) considered *Gemmaria swiftii* Duchassaing de Fonbressin & Michelotti, 1860, to be a species of *Parazoanthus* Haddon & Shackleton, 1891, and transferred this species to the genus. The assignment of *Gemmaria swiftii* Duchassaing de Fonbressin & Michelotti, 1860, to the genus *Parazoanthus* Haddon and Shackleton, 1891, remains the prevailing opinion (e.g. Ryland and Lancaster 2003: 410, Reimer et al. 2014a: 1, 3–7). *Mammillifera clavata* Duchassaing de Fonbressin, 1850, was considered by Reimer et al. (2012a: 8, 9) to be a valid species of *Palythoa* Lamouroux, 1816. The assignment of *Mammillifera brevis* Duchassaing de Fonbressin, 1850, to the genus *Epizoanthus* Gray, 1867, by workers such as Andres (1884: 311) was questioned by Haddon and Shackleton (1891b: 687), who nevertheless stated that “neither *Gemmaria swiftii* nor *Gemmaria brevis* would appear to belong to the same genus as the type species, nor is it certain that *Gemmaria clavata* does either”.

It was probably for these reasons that Haddon and Shackleton (1891a: 626) designated *Gemmaria rusei* Duchassaing de Fonbressin & Michelotti, 1860, as the type species of *Gemmaria* Duchassaing de Fonbressin & Michelotti, 1860. The spelling of the type species, *Gemmaria rusei* Duchassaing de Fonbressin & Michelotti, 1860, requires some discussion. The International Commission on Zoological Nomenclature (ICZN 1998: 121, 122) ruled that the spellings of two genus- and species-group names derived from the surname Riise but earlier incorrectly spelled as Rüsei by Duchassaing de Fonbressin and Michelotti (see Bayer and Grasshoff 1997: 11) should be corrected. However, *Gemmaria rusei* Duchassaing de Fonbressin & Michelotti, 1860, was not one of the names that was ruled on. The spelling of the type species of *Gemmaria* Duchassaing de Fonbressin & Michelotti, 1860, should thus remain as *Gemmaria rusei* Duchassaing de Fonbressin & Michelotti, 1860 (but see also Ryland and Lancester 2003: 410).

The genus-group name *Gemmaria* Duchassaing de Fonbressin & Michelotti, 1860, is however not a valid name as it is a junior homonym of *Gemmaria* McCrady, 1859. The genus-group name *Gemmaria* was conditionally proposed by McCrady (1859: 151) for a new species of hydrozoan, *Zanclea gemmosa*, described in the same paper. Although the genus-group name *Gemmaria* McCrady, 1859, is currently considered to be a junior synonym of *Zanclea* Gegenbaur, 1856 (see Schuchert 2010: 487), it nevertheless remains an available name. *Gemmaria* Duchassaing de Fonbressin & Michelotti, 1860, therefore remains an invalid name as it is preoccupied by *Gemmaria* McCrady, 1859 (Article 53.2 of the Code, ICZN 1999: 57).

Realising this homonymy, Verrill (1900: 562) wrote: “*Protopalythoa* nom. nov. Type *Gemmaria variabilis* Duerden. *Gemmaria* Duch. and Mich., Corall. Antill., p. 55, 1860, (non McCready [*sic*], 1859)”. As *Protopalythoa* Verrill, 1900, was explicitly proposed as a replacement name for *Gemmaria* Duchassaing de Fonbressin & Michelotti, 1860 (preoccupied by *Gemmaria* McCrady, 1859), the designation of *Gemmaria variabilis* Duerden, 1898, as the type species of *Protopalythoa* Verrill, 1900, is invalid.

Article 67.8 of the Code (ICZN 1999: 68) states that “[i]f an author publishes a new genus-group name expressly as a new replacement name (*nomen novum*) for a previously established name … both the prior nominal taxon and its replacement have the same type species, and type fixation for either applies also to the other, despite any statement to the contrary”.

The type species of *Protopalythoa* Verrill, 1900, is therefore *Gemmaria rusei* Duchassaing de Fonbressin & Michelotti, 1860, as this is the type species of *Gemmaria* Duchassaing de Fonbressin & Michelotti, 1860 (as designated by Haddon and Shackleton 1891a: 626). *Protopalythoa* Verrill, 1900, is therefore an objective synonym of *Gemmaria* Duchassaing de Fonbressin & Michelotti, 1860.

In their important paper on *Protopalythoa* Verrill, 1900, Ryland and Lancaster (2003: 410, 411) followed Verrill’s (1900: 562) incorrect type species designation of *Gemmaria variabilis* Duerden, 1898, as the type species of *Protopalythoa* Verrill, 1900. Ryland and Lancaster (2003: 410, 411) stated: “*Gemmaria* Duchassaing and Michelotti, 1860, was introduced for *Gemmaria Rusei* nov., *Mamillifera clavata* Duchassaing, 1850, *Gemmaria swifti* nov. (= *Parazoanthus swifti*), and *Mammillifera brevis* Duchassaing, 1850, with no designation of type species. *Protopalythoa* Verrill, 1900 was a *nomen novum* because *Gemmaria* was preoccupied by *Gemmaria* McCrady, 1857 (cited as McCready, 1859), a hydroid. Verrill designated *Gemmaria variabilis* Duerden, 1898, as type species, on the grounds that *Gemmaria riisei* (sic: *Gemmaria rusei*) was unrecognisable and Duchassaing & Michelotti’s other species were not congeners”. Ryland and Lancaster (2003: 410, 411) considered the characters exhibited by *Gemmaria variabilis* Duerden, 1898 (and other congeners) to be sufficiently different from *Alcyonium mammillosum* Ellis, in Ellis & Solander, 1786 (an objective synonym of the type species of *Palythoa* Lamouroux, 1816), to require a genus-group of their own, for which they used *Protopalythoa* Verrill, 1900.

But what is the real identity of *Protopalythoa* Verrill, 1900? The identity of the genus-group *Protopalythoa* Verrill, 1900, then rests on the identity of its type species *Gemmaria rusei* Duchassaing de Fonbressin & Michelotti, 1860. Volpi and Benvenuti (2003: 66) listed the existence of a syntype of *Gemmaria rusei* Duchassaing de Fonbressin & Michelotti, 1860, at the Museo di Storia Naturale Università di Firenze (accession number: MZUF 847). Through the assistance of C. Volpi and S. Bambi, we have been able to examine high-resolution photographs of the syntype. The images clearly show a species of *Palythoa* Lamouroux, 1816, based on sampling information (depth, locality, etc.) and heavy sand encrustation of the polyps, and therefore *Gemmaria rusei* Duchassaing de Fonbressin & Michelotti, 1860, is referable to the genus *Palythoa* Lamouroux, 1816, as is currently defined.

Although the specific identity of *Gemmaria rusei* Duchassaing de Fonbressin & Michelotti, 1860, will require further research, we herein assign this species-group taxon to *Palythoa* Lamouroux, 1816. *Protopalythoa* Verrill, 1900, thus becomes a junior subjective synonym of *Palythoa* Lamouroux, 1816. Likewise, the preoccupied genus-group name *Gemmaria* Duchassaing de Fonbressin & Michelotti, 1860, as well as *Parapalythoa* Verrill, 1900 (discussed below), and *Haplotella* Stechow, 1919 (also discussed below), become junior subjective synonyms of *Palythoa* Lamouroux, 1816.

Verrill (1900: 562, 1907: 287) himself had already observed the close affinity between *Protopalythoa* Verrill, 1900, and *Palythoa* Lamouroux, 1816, stating “[t]he name *Gemmaria* having been preoccupied in Hydrozoa, it is necessary to give a new one to this group, if it is to be considered as really distinct from *Palythoa*, from which it seems to differ only in the fact that the zoöids are not united together laterally by coenenchyma, but only by stolons or based expansions. Some species of *Palythoa* are not thus united for more than half their height, or even less, and perhaps future discoveries may show a complete gradation between the two conditions” (Verrill 1900: 562). Similarly, Verrill (1907: 287) also discussed that: “[s]hould they [i.e., the species assigned to *Protopalythoa*] ultimately prove to be identical, it would probably be necessary to unite the genus *Protopalythoa* (= *Gemmaria* of many authors) to *Palythoa* … The only tangible difference between the two genera is the presence in the latter of a thick crust-like coenenchyma, uniting the polyps together laterally. But in this species they are often united for less than half their height”.

Two other genus-group names need to be discussed. *Parapalythoa* Verrill, 1900, is another genus-group name that needs to be discussed in connection with *Gemmaria* Duchassaing de Fonbressin & Michelotti, 1860. Verrill (1900: 560) proposed the name *Parapalythoa* for specimens of “*Gemmaria Rusei*” described by McMurrich (1889: 124, 125) from Bermuda. Verrill (1900: 560) considered the material studied by McMurrich (1889) to be distinct from *Gemmaria rusei* Duchassaing de Fonbressin & Michelotti, 1860, and proposed that they should be called *Parapalythoa heilprini*. Although Verrill (1907: 283) later stated that the genus-group name *Parapalythoa* was an error for *Protopalythoa*, this statement has no bearing on the availability of the genus-group name *Parapalythoa*, and the name remains an available one, with *Parapalythoa heilprini* Verrill, 1900, being the type species by monotypy. Regardless of the validity of *Parapalythoa heilprini* Verrill, 1900, it is clear that Verrill (1900: 560) was intending to establish a new genus-group for a taxon similar to, but distinct from, *Gemmaria rusei* Duchassaing de Fonbressin & Michelotti, 1860. As discussed above, *Gemmaria rusei* Duchassaing de Fonbressin & Michelotti, 1860, is herein considered to be a species of *Palythoa* Lamouroux, 1816, and as the differences in the specimens of “*Gemmaria rusei*” described by McMurrich (1889: 124, 125) not being sufficient for a distinction at the genus-group level, we herein consider *Parapalythoa* Verrill, 1900, to also be a junior subjective synonym of *Palythoa* Lamouroux, 1816.

A final name that needs to be discussed in connection with *Gemmaria* Duchassaing de Fonbressin & Michelotti, 1860, is *Haplotella* Stechow, 1919. Stechow (1919: 853) proposed the name *Haplotella* as a replacement name for *Gemmaria* Duchassaing de Fonbressin & Michelotti, 1860, and has the same type species (see discussion above). *Haplotella* Stechow, 1919, is a junior objective synonym of *Gemmaria* Duchassaing de Fonbressin & Michelotti, 1860, and a junior subjective synonym of *Protopalythoa* Verrill, 1900, and *Parapalythoa* Verrill, 1900. The date of publication of *Haplotella* is conventionally cited as “1920”, but in an abstract to the work that appeared in 1919, the following was stated: “Die Aktiniengattung "*Gemmaria*" Duchassaing et Michelotti 1861 erhält wegen Präokkupation durch ein Hydroidengenus den Namen *Haplotella*” (Stechow 1919: 853). The date of publication of the name *Haplotella* is thus 1919.

In conclusion, the genus-group names *Gemmaria* Duchassaing de Fonbressin & Michelotti, 1860, *Parapalythoa* Verrill, 1900, *Protopalythoa* Verrill, 1900, and *Haplotella* Stechow, 1919, are all herein considered to be synonyms of *Palythoa* Lamouroux, 1816. Furthermore, the genus-group name *Protopalythoa* Verrill, 1900, can no longer be used by authors who consider *Gemmaria variabilis* Duerden, 1898 (and other related species) to be be sufficiently distinct to require a genus-group of its own (i.e. *Protopalythoa* sensu Ryland and Lancaster 2003). To resolve this, a new genus-group name will need to be established.

### *Savalia* Nardo, 1844, is a senior subjective synonym of *Gerardia* Lacaze-Duthiers, 1864, with comments the family-group names Gerardiidae Verrill, 1865, Savagliidae Brook, 1889, and Parazoanthidae Delage and Hérouard, 1901

The family-, genus- and species-group names for the zoantharian living on gorgonians in the Mediterranean area has been a matter of a long-running historical debate (reviewed in in Brook 1889: 79, 80, Bell 1891: 89–91; Carlgren 1895: 319–334, Roche and Tixier-Durivault 1951: 402–409).

At the species-group rank, the first available name to be applied to this taxon was “*Gorgonia Savaglia*” by Bertoloni (1819: 219). The name “savaglia” being the commonly used vernacular name for this animal (e.g., Imperato 1599: 724; Garencieres 1676: 5, Bell 1899: 90). Haime (1849: 225, note) later established “*Leiopathes Lamarcki*” (the species-group name being spelled with only one “i”).

At the genus-group rank, Nardo (1844: 433, 434) and Lacaze-Duthiers (1864a: 87) respectively established the genus-group names *Savalia* (type species *Gorgonia savaglia* Bertoloni, 1819), and *Gerardia* (type species *Leoipathes lamarcki* Haime, 1849). Each species is the type species of its respective genus-group by monotypy (Article 68.3 of the Code, ICZN 1999: 71). As a further complication, the genus-group name *Savaglia* was used by Nardo (1877), who was clearly intending to emend the spelling of *Savalia* Nardo, 1844.

Brook (1889: 79, 80) considered the valid binomial name for the species under consideration to be *Savaglia lamarcki* (Haime, 1849). The reason for for doing was given as: “Lacaze Duthiers was the first to show the true relations of this form, and its difference from the typical Antipathidae. Nardo, in 1843 [*sic*], gave the generic name *Savaglia* to the species described as *La Savaglia* by Donati in 1765, which he says is identical with *Leiopathes lamarcki*, Haime; in this case his name has priority over that of *Gerardia*, instituted by Lacaze Duthiers in 1864. This I gather from a more recent paper; I have not seen the original, and do not know if Nardo gave the species a specific, as well as a generic, name; there is no mention of one in his recent publication. I have, therefore, retained the specific name of Haime. Although it seems highly probable that Nardo’s *Savaglia* is the same as *Gerardia*, Lacaze Duthiers, his description of the polyp does not agree with Lacaze Duthiers’ observations on living specimens. Nardo states that the polyp has only fourteen tentacles, whereas the species in question has twenty-four” (Brook 1889: 79).

Bell (1891: 89–91) took the opposite position and considered the valid binomial name to be *Gerardia savalia* (Bertoloni, 1819). Bell (1891: 89–91) argued that the establishment of the genus-group name *Savalia* in Nardo (1844: 433, 434) was not ‘legitimate’ as “Nardo does not write out the full name or names of the species to be placed in this genus, but it is clear that had he done so he would have then written *Savaglia savaglia* (or *Savalia savalia*). This use of a specific for a generic name has been forbidden by the rules of the British Association. Meanwhile this species had become famous by the researches of Lacaze-Duthiers, who, conferring on it (in 1864) the generic name of *Gerardia*, retained for it the specific name of *lamarcki* given it by Haime (in 1849) when he called it *Leiopathes lamarcki*. … It is clear that there is no escape from the conclusion that the proper specific name of this Coral is *savalia*, and the generic *Gerardia* …”.

In order to resolve the matter of the correct genus- and species- group names, a more detailed analysis of the description given by Nardo (1844: 433, 434). The description is given as follows: “*Sotto famiglia II.^a^ Savalini — Polipi a sedici tentacoli! Gen.* Savalia *N. … Fa meraviglia come sia sfuggito all’occhio deglie osservatori il bel lavoro del Donati V. sull’Antipate dell’ Adriatico* Gorgonia savoglia [*sic*] *(Bertoloni) inserito nel primo volume del Giornale di Grisellino, ove vedesi esattamente descritto e figurato l’animale con i suoi sedici tentacoli. Non v’ha dubbio che una tale specie debbasi distinguere dal genere* Anthipathes. *Costituisee anzi a mio credere una sotto famiglia, come mostreró in più esteso lavoro relativo ai caratteri distintivi delle famiglie dei Zoofitarj*” (Nardo 1844: 433, 434).

This is translated as: “Subfamily 2. Savalini — Polyps with 16 tentacles. Genus *Savalia* N[obis]. … The fine work of V. Donati on Antipate [= black corals/zoophytes] of the Adriatic Sea in the first volume of the *Giornale di Grisellino* has long been overlooked by previous workers, in which *Gorgonia savoglia* [*sic*] (Bertoloni) was exactly described and figured as an animal with sixteen tentacles. There is not doubt that this species is deserving of a genus distinct from the genus *Anthipathes*. Indeed, as I will show in a more extensive work relating to the distinctive characteristics of the families of Zoofitarj, it constitutes a new subfamily”.

The objection raised by Brook (1889: 79) for not using *Gorgonia savaglia* Bertoloni, 1819, as the species-group name is not tenable, as the only reason given is that he did “not know if Nardo gave the species a specific name” as Brook did not have access to the 1844 paper by Nardo. From the description by Nardo (1844: 433, 434), the genus-group name Savalia was clearly established for *Gorgonia savaglia* Bertoloni, 1819 (incorrectly spelled as “*savoglia*”). Virtually all recent authors consider *Gorgonia savaglia* Bertoloni, 1819, to be a subjective synonym of *Leiopathes lamarcki* Haime, 1849 (e.g. Roche and Tixier-Durivault 1951: 402, Ocaña et al. 1995: 155, Ocaña and Brito 2004: 170, Sinniger et al. 2005: 1124, 1125, Ocaña et al. 2007: 163–167). *Gorgonia savaglia* Bertoloni, 1819, has priority over *Leiopathes lamarcki* Haime, 1849, and the former is the valid species-group name.

The objections raised by Bell (1891: 90) for not accepting *Savalia* Nardo, 1844, are also not justified. Firstly, Nardo (1844: 433, 434) did indeed provide the name of the species he intended to be placed in *Savalia* Nardo, 1844 (as discussed above). Secondly, as already stated by Poche 1914: 104, tautomeric names (viz., “*Savaglia savaglia* / *Savalia savalia*”) are not invalid on this basis alone (Articles 18 and 23.3.7 of the Code, ICZN 1999: 21, 26). As their respective type species are considered to be subjective synonyms (see above), the genus-group name *Savalia* Nardo, 1844, is a subjective synonym of *Gerardia* Lacaze-Duthiers, 1864 (see also Sinniger et al. 2013: [1], [2], [7]). As *Savalia* Nardo, 1844, has priority over *Gerardia* Lacaze-Duthiers, 1864, the former is the valid genus-group name.

At the family-group rank, three names have been proposed for *Savalia savaglia* (Bertoloni, 1819). As noted above, Nardo (1844: 433, 434) established the family-group name Savaliidae (as “Savalini”) based on the genus-group name *Savalia* Nardo, 1844. Later, Verrill (1869 [in 1868–1870]: 499) established the family-group name Gerardiidae (as “Gerardiidae”). Brook (1889: 79) wrote “Savagliidae, n[om]. n[ov]. (Gerardiidae [*sic*], Verrill)”. Brook (1889: 79, 80) based this family-group name based on the emended name *Savaglia* Nardo, 1877, as he did not have access to Nardo (1844) (discussed above). The family-group name Savagliidae proposed by Brook (1889: 79) is herein regarded to have been proposed independently of Savaliidae Nardo, 1844 (based on *Savalia* Nardo, 1844). As their respective type genera are synonyms (as discussed above), the family-group names Savaliidae Nardo, 1844, Gerardiidae Verrill, 1865, and Savagliidae Brook, 1889, are all synonyms.

The oldest and therefore valid family-group name for *Savalia savaglia* (Bertoloni, 1819) is therefore Savaliidae Nardo, 1844. The type genus of Savaliidae Nardo, 1844, (i.e., *Savalia* Nardo, 1844), is however currently assigned to the family Parazoanthidae Delage & Hérouard, 1901 (Sinniger et al. 2013: [2], [3]). This means that the family-group names Savaliidae Nardo, 1844, Gerardiidae Verrill, 1865, and Savagliidae Brook, 1889, are all subjective synonyms of Parazoanthidae Delage & Hérouard, 1901. To maintain current and widespread use of Parazoanthidae Delage & Hérouard, 1901, the best course of action would be to enact Article 23.9 of the Code (ICZN 1999: 27, 28) to reverse precedence between Parazoanthidae Delage & Hérouard, 1901, and the other three family-group names. This is not possible, however, as all three family-group names have been used after 1899, and Article 23.9.1.1 of the Code (ICZN 1999: 28) cannot be satisfied.

In accordance with Article 23.9.2 (ICZN 1999: 28, 29), an application is being prepared to request that the International Commission on Zoological Nomenclature suppress the senior subjective synonyms Savaliidae Nardo, 1844, Gerardiidae Verrill, 1865, and Savagliidae Brook, 1889, in favour of Parazoanthidae Delage & Hérouard, 1901, to maintain current and widespread usage.

### Sphenopidae Hertwig, 1882, and the hitherto overlooked subjective synonym Palythoidae Duchassaing de Fonbressin and Michelotti, 1860

The family-group name Sphenopidae was established by Hertwig (1882: 120) for the genus *Sphenopus* Steenstrup, 1856. The name Sphenopidae Hertwig, 1882, had been used sporadically since it was established. Since it was reinstated by Ryland et al. (2000: 191, 192) for the genera *Sphenopus* Steenstrup, 1856, and *Palythoa* Lamouroux, 1816, however, the name Sphenopidae Hertwig, 1882, is now in current and widespread use (see references cited below).

This current and widespread use of the family-group name Sphenopidae Hertwig, 1882, is threatened by the hitherto overlooked subjective synonym Palythoidae Duchassaing de Fonbressin & Michelotti, 1860 (type genus *Palythoa* Lamouroux, 1816).

This family-group name was first established as “Polythoae” for “*Polythoa*”, “*Bergia* n. g.” and “*Gemmaria* n. g.” (Duchassaing de Fonbressin & Michelotti, 1860: 37). That Duchassaing de Fonbressin and Michelotti (1860: 37) intended this to be a family-group name is evidenced by their use of “Zoanthidae” (containing “*Zoanthus*”, “*Isaura*”, “*Mammillifera*” and “*Orinia* n. g.”) as a counterpart to “Polythoae”. The name “*Polythoa*” is an incorrect subsequent spelling of *Palythoa* Lamouroux, 1816 (the first usage of which appears to be by Schweigger 1819: 100).

As type genus of Sphenopidae Hertwig, 1882, and of Palythoidae Duchassaing de Fonbressin & Michelotti, 1860, are considered to belong to the same family grouping (e.g., Ryland et al. 2000: 192; Reimer et al. 2012b: 45, 47, 49; Irei et al. 2015: 2, 3, 14–16; Reimer and Fujii 2016: 14, 19), both family-group names are therefore subjective synonyms.

To prevent the nomenclatural and taxonomic destabilisation that would result from the replacement of Sphenopidae Hertwig, 1882, with its subjective synonym Palythoidae Duchassaing de Fonbressin and Michelotti, 1860, as required by the Principle of Priority (Article 23, ICZN 1999: 24) requires that the oldest available name for the taxon under consideration must be used.

To mediate the Principle of Priority, Article 23.9.1 of the Code (ICZN 1999: 27) allows for a reversal of precedence of a junior synonym when the senior synonym has not been used as a valid name after 1899 (Article 23.9.1.1) and the junior synonym “has been used for a particular taxon, as its presumed valid name, in at least 25 works, published by at least 10 authors in the immediately preceding 50 years and encompassing a span of not less than 10 years” (Article 23.9.1.2).

Since 1899, the name Palythoidae has been used in three publications. The first usage was by Barel and Kramers (1977: 32) used the term “species of Palythoidae” for an unidentified zoantharian associated with echinoderms. From the context, it appears that Barel and Kramers (1977: 32) were reporting on material with close affinities to *Palythoa* Lamouroux, 1816.

As Ng and Low (2010:37, 38) have argued, “valid usage” of a name must be unambiguous and show clearly that the author[s] both considered it the correct name to be used and adopted the name. According to these criteria, the usage of “Palythoidae” by Barel and Kramers (1977: 32) cannot be considered to be valid.

The second usage was by Herberts (1972: 125) who stated: “La disposition brachynémique des mésentères est un autre caractère des Palythoidae”. That Herberts (1972: 125) was not using “Palythoidae” as a valid family-group name is evidenced by the fact that the section in which the term “Palythoidae” appears is headed by “Zoanthidae” (p. 106), and in a diagram detailing the classification of zoantharians, “*Palythoa*”, “*Isaurus*”, “*Spenopus* [*sic*]” and “*Zoanthus*” are clearly placed in “Zoanthidae” (p. 142).

The third usage is by Pax and Müller (1957: 3, 4) in which they recognise the “Unterfamilie Palythoinae” under Epizoanthidae, and further provide a key for identifying this subfamily. Clearly, Pax and Müller (1957: 3, 4) were recognising the family-group name Palythoidae as vaild.

The name Palythoidae Duchassaing de Fonbressin and Michelotti, 1860, thus has been used as the valid name for the taxon is denotes since 1899 (and Article 23.9.1.1 of the Code cannot be fulfulled).

Article 23.9.1.2 of the Code is fulfilled as the family-group name Sphenopidae Hertwig, 1882, is in current and widespread usage, as evidenced by the 30 publications by 99 different authors over the past 34 years using Sphenopidae as a valid name for the taxon it denotes (viz., Nagabhushanam and Jothinayagam 1982: 17; Ryland et al. 2000: 191, 192; Ryland and Lancaster 2003: 407, 409, 415; Ryland and Lancaster 2004: 180; Ryland and Westphalen 2004: 411; Ryland et al. 2004: 1195, 1197; Acosta et al. 2005: 147–149, 151, 154, 160; Sinniger et al. 2005: 1122, 1125, 1126; Daly et al. 2007: 144; Sinniger et al. 2008: 1254, 1256, 1257; Fautin and Daly 2009: 356; Del Mónaco et al. 2010: 360; Reimer and Sinniger 2010: 251; Reimer et al. 2010c: 606, 616; Swain 2010: 2592; Reimer et al. 2011a: 983, 985, 987, 989, 991, 992; Cavallari et al. 2012: 25; Longo et al. 2012: [1]; Palmer et al. 2012: 3880; Reimer et al. 2012b: 43, 45, 47, 49; Rodríguez-Viera et al. 2012: 32; Costello et al. 2013: [2]; Fujii and Reimer 2013: 510, 516; Krishna and Gophane 2013: 210; Koupaei et al. 2014: 64; Alencar et al. 2015: 1113, 1114, 1121; Irei et al. 2015: 1, 2, 4, 6, 14–16, 20; Qin et al. 2015: 100; De la Cruz-Francisco et al. 2016: 24; Fujii and Reimer 2016: 11, 12, 14, 17, 19, 20; Risi and Macdonald 2016: 113).

As precedence of the family-group names Sphenopidae Hertwig, 1882, and Palythoidae Duchassaing de Fonbressin & Michelotti, 1860, cannot be replaced, the former will need to be replaced by the latter. To prevent resulting nomenclatural instability, and in accordance with Article 23.9.2 (ICZN 1999: 28, 29), an application is being prepared to request that the International Commission on Zoological Nomenclature suppress the senior subjective synonym Palythoidae Duchassaing de Fonbressin & Michelotti, 1860, in favour of Sphenopidae Hertwig, 1882, to maintain current and widespread usage.

### The type species of *Palythoa* Lamouroux, 1816

The genus-group name *Palythoa* (with the simultaneous French vernacular ‘Palythoé’) was established by Lamouroux (1816: 359–362) with the inclusion of two species, which were rendered French vernacular: ‘Palythoé Etoilée’ and ‘Palythoé Ocellée’. The second species causes no problems “*Palythoa Ocellata* … Sol. et Ell., p. 180, n. 6, tab. 1, fig. 6 (*A. Ocellatum*) …” is listed. This is *Alcyonium ocellatum* Ellis, in Ellis and Solander, 1786, a synonym of *Alcyonium mammillosum* Ellis, in Ellis and Solander, 1786 (see Pax 1910a: 102; 1910b: 258; Acosta et al. 2005: 159). The French vernacular name ‘Palythoé Etoilée’, however, is not the translation of the name “*P*[*alythoa*] *Mamillosa* [*sic*] … Sol. et Ell., p. 179, n. 5, tab. 1, figs. 4–5 (*A. Mamillosum* [*sic*]) …” given in the synonymy.

On an unnumbered page of errata after page 559 of Lamouroux (1816) gives “[page] 361 … [line] 12 … *Mamillosa*, lisez *Stellata*”, and the captions on page 558 to plate 14 (and on the plate itself) of Lamouroux (1816) gives “*Palythoa* [sic] *stellata*, p. 361. figure copiée dans Ellis”. It is thus clear that it was the intention of Lamouroux (1816: 361, 558, unnumbered errata page, pl. 14, fig. 2, caption) to rename *Alcyonium mammillosum* Ellis, in Ellis & Solander, 1786, *Palythoa stellata*. *Palythoa* [*sic*] *stellata* Lamouroux, 1816, is also a junior objective synonym of *Alcyonium mammillosum* Ellis, in Ellis & Solander, 1786, as Lamouroux (1816: 361, 558, unnumbered errata page, pl. 14, fig. 2, caption) proposed the former as a replacement name for the latter.

*Alcyonium ocellatum* Ellis, in Ellis & Solander, 1786, and *Palythoa stellata* Lamouroux, 1816, are thus to be considered species originally included in the genus *Palythoa* Lamouroux, 1816, and were both eligible for fixation (Article 67.2 of the Code, ICZN 1999: 67).

The type species of the genus *Palythoa* Lamouroux, 1816, is accepted as *Alcyonium mammillosum* Ellis, in Ellis & Solander, 1786, by subsequent designation by Haddon and Shackleton (1891b: 691) (see Low and Reimer 2011b: 63). As Lamouroux (1816: 361, 558, unnumbered errata page, pl. 14, fig. 2, caption) proposed the replacement name *Palythoa stellata* for *Alcyonium mammillosum* Ellis, in Ellis & Solander, 1786, the latter cannot be considered an originally included species in the sense of Article 67.2 of the Code (ICZN 1999: 67).

Article 69.2.2 of the Code (ICZN 1999: 72) states that “[i]f an author designates as type species a nominal species that was not originally included (or accepts another’s such designation) and if, but only if, at the same time he or she places that nominal species in synonymy with one and only one of the originally included species (as defined in Article 67.2), that act constitutes fixation of the latter species as type species of the nominal genus or subgenus”.

Haddon and Shackleton (1891b: 691) wrote that “*Palythoa mammillosa* is evidently regarded by Lamouroux [1816] as the type species of the genus. He reproduces Solander’s figure of this species, but not that of *Palythoa ocellata*, of which he merely gives a description. Unfortunately a Latinized version of the French name ‘*Palythoé Etoillée*,’ given by Lamouroux to *Palythoa mammillosa*, has been added at the bottom of his plate—a circumstance which has given rise to some confusion”.

Clearly, Haddon and Shackleton (1891b: 691) considered *Alcyonium mammillosum* Ellis, in Ellis & Solander, 1786, to be a synonym of *Palythoa* [*sic*] *stellata* Lamouroux, 1816, as well as the type species of *Palythoa* Lamouroux, 1816. The conditions of Article 69.2.2 of the Code (ICZN 1999: 72) are met and Haddon and Shackleton (1891b: 691) are deemed to have designated *Palythoa* [*sic*] *stellata* Lamouroux, 1816, as the type species of *Palythoa* Lamouroux, 1816. As both names are objective synonyms, the valid name for the species under discussion is *Palythoa mammillosa* (Ellis, in Ellis & Solander, 1786).

**Table 1.** An updated supraspecific classification of the Zoantharia. All valid genera and their type species are given. The numbers given in parentheses after each suborder and family represent the total number of families and genera, respectively, in each grouping. A total of one order, three suborders, nine families and twenty-seven genera are currently recognised in the Zoantharia.

**Order Zoantharia Rafinesque, 1815**
**1. Suborder Brachycnemina Haddon AND Shackleton, 1891 (3)**
**1. Neozoanthidae Herberts, 1972 (1)**
1. *Neozoanthus* Herberts, 1972
*Neozoanthus tulearensis* Herberts, 1972
**2. Sphenopidae Hertwig, 1882 (2)**
2. *Sphenopus* Steenstrup, 1856
*Sabella marsupialis* Gmelin, 1791
3. *Palythoa* Lamouroux, 1816
*Palythoa* [*sic*] *stellata* Lamouroux, 1816
**3. Zoanthidae Rafinesque, 1815 (3)**
4. *Zoanthus* Lamarck, 1801
*Actinia sociata* Ellis, 1768
5. *Acrozoanthus* Saville-Kent, 1893
*Acrozoanthus australiae* Saville-Kent, 1893
6. *Isaurus* Gray, 1828
*Isaurus tuberculatus* Gray, 1828
**2. Suborder Macrocnemina Haddon & Shackleton, 1891 (5)**
**4. Epizoanthidae Delage & Hérouard, 1901 (3)**
7. *Epizoanthus* Gray, 1867
*Dysidea papillosa* Johnston, 1842
8. *Paleozoanthus* Carlgren, 1924
*Paleozoanthus reticulatus* Carlgren, 1924
9. *Thoracactis* Gravier, 1918
*Thoracactis topsenti* Gravier, 1918
**5. Hydrozoanthidae Sinniger, Reimer & Pawlowski, 2010 (2)**
10. *Hydrozoanthus* Sinniger, Reimer & Pawlowski, 2010
*Parazoanthus tunicans* Duerden, 1900
11. *Terrazoanthus* Reimer & Fujii, 2010
*Terrazoanthus onoi* Reimer & Fujii, 2010
**6. Microzoanthidae Fujii & Reimer, 2011 (1)**
12. *Microzoanthus* Fujii & Reimer, 2011
*Microzoanthus occultus* Fujii & Reimer, 2011
**7. Nanozoanthidae Fujii & Reimer, 2013 (1)**
13. *Nanozoanthus* Fujii & Reimer, 2013
*Nanozoanthus harenaceus* Fujii & Reimer, 2013
**8. Parazoanthidae Delage & Hérouard, 1901 (13)**
14. *Parazoanthus* Haddon & Shackelton, 1891
*Palythoa axinella* Haddon & Shackelton, 1891
15. *Antipathozoanthus* Sinniger, Reimer & Pawlowski, 2010
*Gerardia macaronesicus* Ocaña & Brito, 2003
16. *Bergia* Duchassaing de Fonbressin & Michelotti, 1860
*Bergia catenularis* Duchassaing de Fonbressin & Michelotti, 1860
17. *Bullagummizoanthus* Sinniger, Ocaña & Baco, 2013
*Bullagummizoanthus emilyacardiarum* Sinniger, Ocaña & Baco, 2013
18. *Corallizoanthus* Reimer, *in* Reimer, Nonaka, Sinniger & Iwase, 2008
*Corallizoanthus tsukaharai* Reimer, *in* Reimer, Nonaka, Sinniger & Iwase, 2008
19. *Hurlizoanthus* Sinniger, Ocaña & Baco, 2013
*Hurlizoanthus parrishii* Sinniger, Ocaña & Baco, 2013
20. *Isozoanthus* Carlgren, in Chun, 1903
*Isozoanthus giganteus* Carlgren, in Chun, 1903
21. *Kauluzoanthus* Sinniger, Ocaña and Baco, 2013
*Kauluzoanthus kerbyii* Sinniger, Ocaña & Baco, 2013
22. *Kulamanamana* Sinniger, Ocaña and Baco, 2013
*Kulamanamana haumeaae* Sinniger, Ocaña & Baco, 2013
23. *Mesozoanthus* Sinniger and Häussermann, 2009
*Mesozoanthus fossii* Sinniger and Häussermann, 2009
24. *Savalia* Nardo, 1844
*Gorgonia savaglia* Bertolini, 1819
25. *Umimayanthus* Montenegro, Sinniger & Reimer, 2015
*Umimayanthus chanpuru* Montenegro, Sinniger & Reimer, 2015
26. *Zibrowius* Sinniger, Ocaña & Baco, 2013
*Zibrowius ammophilus* Sinniger, Ocaña & Baco, 2013
**3. Suborder *incerta sedis* (1)**
**9. Abyssoanthidae Reimer & Fujiwara, in Reimer, Sinniger, Fujiwara, Hirano & Maruyama, 2007 (1)**
27. *Abyssoanthus* Reimer & Fujiwara, *in* Reimer, Sinniger, Fujiwara, Hirano & Maruyama, 2007
*Abyssoanthus nankaiensis* Reimer & Fujiwara, *in* Reimer, Sinniger, Fujiwara, Hirano & Maruyama, 2007
